# Mitochondrial supplementation of *Sus scrofa* metaphase II oocytes alters DNA methylation and gene expression profiles of blastocysts

**DOI:** 10.1186/s13072-022-00442-x

**Published:** 2022-04-15

**Authors:** Takashi Okada, Stephen McIlfatrick, Nhi Hin, Nader Aryamanesh, James Breen, Justin C. St. John

**Affiliations:** 1 Mitochondrial Genetics Group, Robinson Research Institute, School of Biomedicine, Faculty of Health and Medical Sciences, The University of Adelaide, Adelaide, SA 5000 Australia; 2 South Australian Genomics Centre, South Australian Health and Medical Research Institute, SAHMRI, Adelaide, SA 5000 Australia; 3 Present Address: Embryology Research Unit, Bioinformatics Group, Children’s Medical Research Institute, University of Sydney, Westmead, NSW 2145 Australia

**Keywords:** Mitochondrial DNA, DNA methylation, Mitochondrial supplementation, Assisted reproductive technology, Blastocyst, Oocyte, *Sus scrofa*, Whole genome bisulfite sequencing, Transcriptome analysis

## Abstract

**Background:**

Mitochondrial DNA (mtDNA) copy number in oocytes correlates with oocyte quality and fertilisation outcome. The introduction of additional copies of mtDNA through mitochondrial supplementation of mtDNA-deficient *Sus scrofa* oocytes resulted in: (1) improved rates of fertilisation; (2) increased mtDNA copy number in the 2-cell stage embryo; and (3) improved development of the embryo to the blastocyst stage. Furthermore, a subset of genes showed changes in gene expression. However, it is still unknown if mitochondrial supplementation alters global and local DNA methylation patterns during early development.

**Results:**

We generated a series of embryos in a model animal, *Sus scrofa*, by intracytoplasmic sperm injection (ICSI) and mitochondrial supplementation in combination with ICSI (mICSI). The DNA methylation status of ICSI- and mICSI-derived blastocysts was analysed by whole genome bisulfite sequencing. At a global level, the additional copies of mtDNA did not affect nuclear DNA methylation profiles of blastocysts, though over 2000 local genomic regions exhibited differential levels of DNA methylation. In terms of the imprinted genes, DNA methylation patterns were conserved in putative imprint control regions; and the gene expression profile of these genes and genes involved in embryonic genome activation were not affected by mitochondrial supplementation. However, 52 genes showed significant differences in expression as demonstrated by RNAseq analysis. The affected gene networks involved haematological system development and function, tissue morphology and cell cycle. Furthermore, seven mtDNA-encoded t-RNAs were downregulated in mICSI-derived blastocysts suggesting that extra copies of mtDNA affected tRNA processing and/or turnover, hence protein synthesis in blastocysts. We also showed a potential association between differentially methylated regions and changes in expression for 55 genes due to mitochondrial supplementation.

**Conclusions:**

The addition of just an extra ~ 800 copies of mtDNA into oocytes can have a significant impact on both gene expression and DNA methylation profiles in *Sus scrofa* blastocysts by altering the epigenetic programming established during oogenesis. Some of these changes may affect specific tissue-types later in life. Consequently, it is important to determine the longitudinal effect of these molecular changes on growth and development before considering human clinical practice.

**Supplementary Information:**

The online version contains supplementary material available at 10.1186/s13072-022-00442-x.

## Background

Infertility has been an increasing problem in developing countries for the last few decades, due to modern lifestyle patterns, unbalanced diets, and later life stage pregnancies, amongst other factors [1, 2]. In the context of female fertility, oocyte quality declines with advancing age [3], and it has been shown in human and other mammalian species that oocyte mitochondrial DNA (mtDNA) copy number negatively correlates with aging [4–6]. Generally, in mature, fertile oocytes, there are > 200,000 copies of mtDNA present and significant correlations between mtDNA copy number and fertilisation outcome have been reported in studies from human assisted reproductive technology clinics, indicating an association between mtDNA copy number and oocyte quality [7–9]. As a result, several clinics worldwide have sought to introduce mitochondrial supplementation protocols into clinical practice without understanding the consequences of such actions at a molecular level.

mtDNA copy number is strictly regulated during oocyte development. The primordial germ cells, the first germ cells that are laid down, possess ~ 1500 copies of mtDNA per cell [10]. These copies are exponentially replicated during oogenesis ensuring sufficient copies of mtDNA are available at fertilisation and to support subsequent developmental events [11]. Indeed, the mtDNA present in the mature, metaphase II oocyte is an important investment in subsequent developmental outcomes as there are no major mtDNA replication events in cells giving rise to the embryo proper until post-gastrulation [12, 13]. However, replication does take place in the trophectodermal cells, which give rise to the placenta, from the blastocyst stage onwards [14]. As a result, mtDNA copy number decreases in each newly formed cell by half due to cell division coupled with the likely extrusion of mtDNA from the embryo into its neighbouring environment [15]. Consequently, mature oocytes with insufficient copies of mtDNA (< 100,000 copies) would likely have too few copies [7] to promote development to the post-gastrulation stages when mtDNA replication is initiated [12, 13].

mtDNA deficiency can be overcome by supplementing oocytes with additional copies of mtDNA at the time of fertilisation. This results in improved rates of fertilisation and development of the embryo to the blastocyst stage, as demonstrated in a pig (*Sus scrofa*) model [16]. Although only ~ 800 copies of mtDNA were introduced into mtDNA-deficient *Sus scrofa* oocytes, which represents less than 1% of oocyte mtDNA copy number, a mtDNA replication event was induced, which increased mtDNA copy number by 4.4-fold by the 2-cell stage of embryo development [16]. Induction of early mtDNA replication in mtDNA-deficient oocytes could improve embryo quality by stabilising the embryo prior to embryonic genome activation (EGA) [16]. mtDNA replication is controlled by a number of genes encoded by the nuclear genome and many of these factors are unique to mitochondrial replication, and one of the key factors is DNA polymerase gamma (POLG) [12]. *POLG* is DNA methylated in a CpG island at exon 2 and methylation levels negatively correlated with mtDNA copy number in cancer and stem cells [17, 18]. Furthermore, mitochondrial supplementation modulated the methylation status of *POLG*, resulting in a significant and negative correlation between *POLG* methylation and mtDNA copy number in mtDNA-deficient oocytes and developing embryonic cells [19]. Transcriptome analysis also revealed reduced gene expression associated with metabolic disorders in blastocysts derived from supplementation of mtDNA-deficient porcine oocytes [16].

Interestingly, a transgenerational study in mice derived from mtDNA-supplemented oocytes revealed a significant increase in litter size and the number of primordial follicles across three generations, supported by changes in gene expression in primordial follicles [20]. However, it also showed a defect in cardiac structure in first- and second-generation offspring. Consequently, mitochondrial supplementation could lead to modulation of nuclear gene expression profiles and alter epigenetic patterns which may have transgenerational effects. As a result, the interactions that were established between the two genomes throughout oogenesis, namely their 'Genomic Balance', could be perturbed through supplementation which could have downstream implications for cellular function and, ultimately, offspring health and well-being [11, 21].

Since it is still largely unknown if mitochondrial supplementation alters global DNA methylation patterns during early development, we investigated the DNA methylation status of *Sus scrofa* blastocysts derived through mtDNA supplementation by whole genome bisulfite sequencing (WGBS). We also analysed the gene expression profiles of the same stage blastocysts by RNAseq and integrated the two data sets to determine the degree of overlap between changes in DNA methylation and gene expression. To this extent, we used in vitro matured metaphase II oocytes in order to directly address the impact of solely adding extra copies of mtDNA into oocytes. We chose to model these events in the pig as it is regarded as an excellent model to study human pathophysiology [22]. Many of its organ systems and physiological and pathophysiological responses are similar to those of the human, including oocyte and embryo development [23], and it shares similar patterns of mtDNA replication [9, 14, 16]. Furthermore, epigenetic reprograming processes and gene expression profiles during early embryogenesis are conserved between human and pig [24, 25].

We identified > 2000 differentially methylated regions (DMRs) and 52 differentially expressed genes (DEGs) between supplemented and non-supplemented derived blastocysts; and documented regions of common overlap. These outcomes indicate that the addition of just an extra ~ 800 copies of mtDNA can have a significant impact on both gene expression and DNA methylation profiles in *Sus scrofa* blastocysts. They also highlight the importance of the synergy that is established between the two genomes during oogenesis and the potential cost of perturbing these interactions in the metaphase II oocyte.

## Results

### WGBS of *Sus scrofa* ICSI- and mICSI-derived blastocysts

**Fig. 1:**
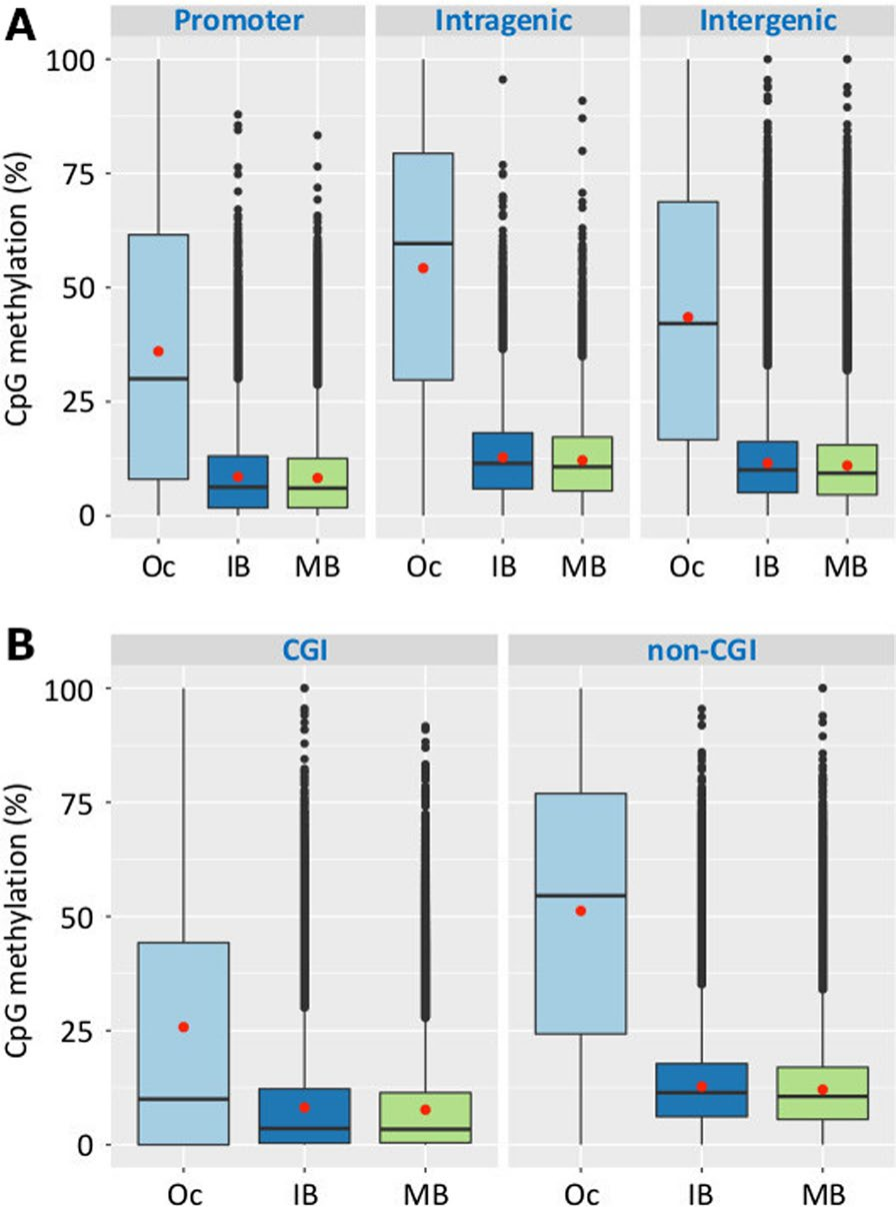
Global DNA methylation levels for *Sus scrofa* oocyte (Oc), ICSI- (IB) and mICSI- (MB) derived blastocysts for various genomic regions. **A** Levels of CpG methylation in promoter, intragenic and intergenic regions are displayed by box plots. **B** Levels of CpG methylation in CpG islands (CGI) and non-CGI regions. Red dots represent the mean value for each group and the black dots indicate outliers

In order to investigate the effects of introducing extra copies of mtDNA into in vitro matured *Sus scrofa* oocytes on global DNA methylation, we assessed blastocysts that were generated through ICSI and mICSI. To this effect, we performed WGBS on pooled populations of non-supplemented oocytes (*n* = 40–63) and expanded blastocysts (*n* = 6), which had undergone DNA extraction and WGBS library preparation. For mICSI-derived blastocysts, mitochondria isolated from sister oocytes were used for autologous supplementation to avoid mitochondrial heterogeneity and genetic complexity (Additional file 1: Fig. S1 and Methods). WGBS libraries were sequenced using the Illumina NovaSeq instrument and 300 to 525 million paired-end reads were obtained per sample (Additional file 2: Table S1). In total, 20 to 75 million reads per sample were uniquely mapped to the *Sus scrofa* genome assembly v11.1 after quality filtering of sequence reads, alignment and removal of duplicated reads by *Bismark* [26]. The mapped reads were then used to make DNA methylation calls in the CpG context. In all, 10 to 32 million CpG sites (17–53%) throughout the *Sus scrofa* genome were covered by at least one read in each data set. Unbiased methylation analysis using the 100-CpG probe method [27] was used to analyse the methylation status for various genomic features (promoters; intragenic and intergenic regions and CpG islands). Our data generated a total of 528,995 100-CpG windows throughout the *Sus scrofa* genome (Additional file 1: Fig. S2), which is about 80% more than previously reported [28], indicating higher coverage and density of CpGs analysed in this study. Correlation analysis of each WGBS data set revealed close association within the same blastocyst type and clear distinctions between oocyte and blastocyst data sets (Additional file 1: Fig. S3A). Overall levels of DNA methylation (Additional file 2: Table S1) were higher in oocytes (> 39% in the CpG context) than blastocysts (< 14%), consistent with previous findings and confirming that DNA demethylation and epigenetic reprogramming are dynamic processes that take place following fertilisation and during preimplantation embryo development in the pig [28–30]. We also confirmed that levels of DNA methylation were higher in intragenic regions and lower in promoter and intergenic regions and CpG islands (CGI; Fig. 1) [31]. Therefore, the WGBS data obtained from *Sus scrofa* ICSI- and mICSI-derived blastocysts had sufficient coverage and CpG site density and exhibited the typical patterns of DNA methylation expected of mammalian blastocysts.

### Global DNA methylation patterns in ICSI- and mICSI-derived blastocysts

**Fig. 2:**
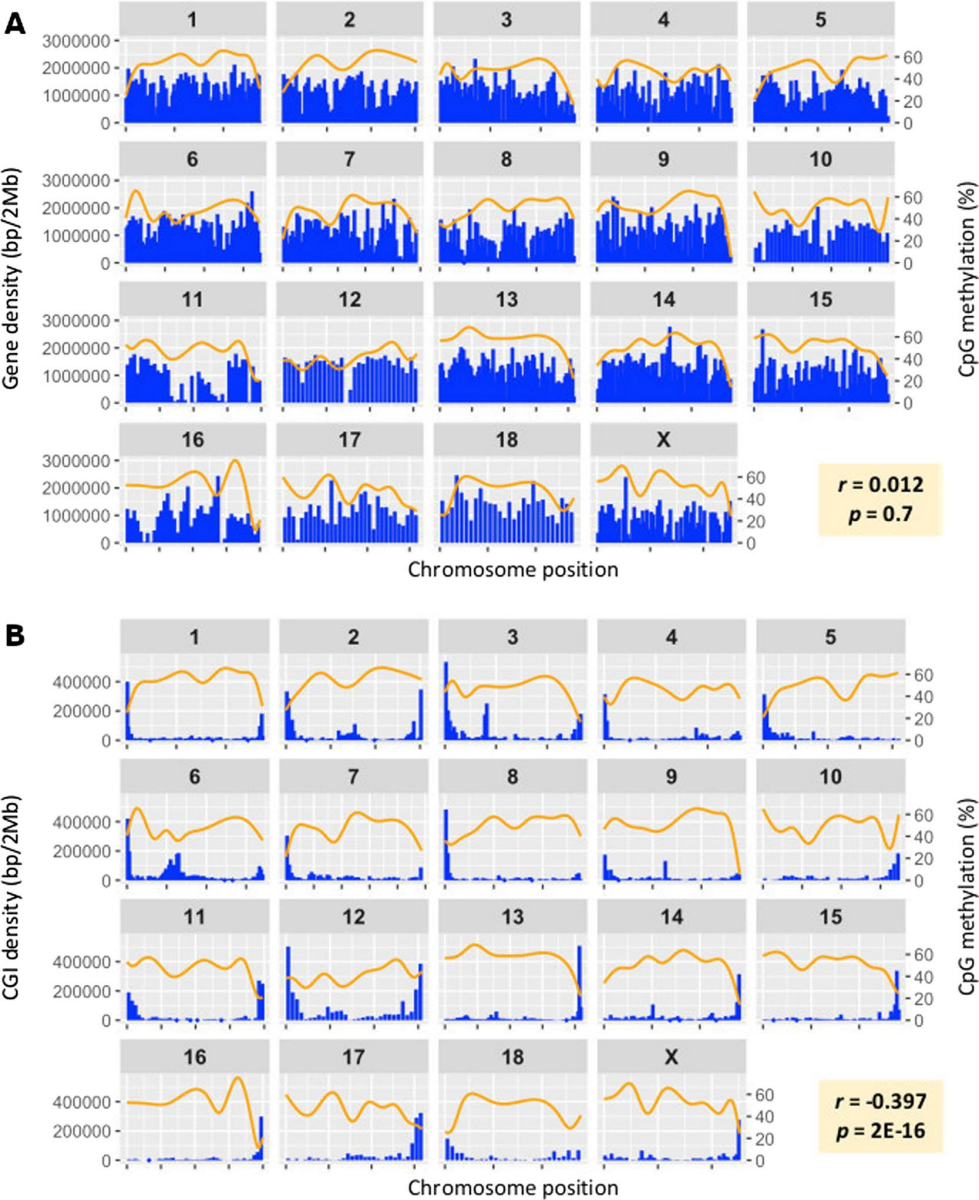
Association between levels of DNA methylation and genomic regions of interest in *Sus scrofa* oocytes. **A** Correlation between CpG methylation and gene density at the chromosomal level. **B** Correlation between CpG methylation and CGI density at the chromosomal level. Orange lines represent smoothed CpG methylation (%) and blue bars indicate genomic feature (CGI or gene) density calculated by bp length in 2 Mbp bins. Pearson's correlation coefficient (*r*) and associated *p*-values are shown at the bottom right

We examined if there were significant differences in DNA methylation patterns between ICSI- and mICSI-derived blastocysts at a global level. Firstly, we investigated individual WGBS data sets to determine if there were any significant variations at a batch level. Both hierarchical clustering and principal component analysis (PCA) showed no apparent differences as they closely clustered together and were separated from oocytes (Additional file 1: Fig. S3B and C). Next, we examined the methylation status at a chromosomal level. In oocytes, we found that the levels of DNA methylation tended to be lower at the distal ends of many chromosomes (Additional file 1: Fig. S4A). As transposon and gene density varies amongst chromosomal regions and associates with levels of methylation within chromosomal regions in plants [32], we investigated this in *Sus scrofa* oocytes. Correlation analysis did not reveal any significant association between methylation levels and gene density in our WGBS data sets (Fig. 2A). However, the density of CGIs significantly and negatively correlated (*r* = − 0.397) with regional methylation levels in *Sus scrofa* chromosomes (Fig. 2B). Similar patterns but lower levels of methylation were found in ICSI- and mICSI-derived blastocysts and correlation levels were also lower (*r* = − 0.079 and − 0.109 for ICSI- and mICSI-derived blastocysts, respectively) (Additional file 1: Fig. S4B and C). Overall mean levels of CpG methylation were slightly higher in ICSI-derived blastocysts compared to mICSI-derived blastocysts, but not significantly (Additional file 2: Table S1). Furthermore, ICSI- and mICSI-derived blastocysts did not show obvious differences in CpG methylation patterns at a chromosomal level (Additional file 1: Fig. S4). Therefore, at a global level, mitochondrial supplementation did not affect nuclear DNA methylation patterns in blastocysts.

### Differentially methylated regions (DMRs) between ICSI- and mICSI-derived blastocysts

Although there were no apparent differences in global DNA methylation patterns between ICSI- and mICSI-derived blastocysts, it is conceivable that there was a number of DMRs present at a local level. There are several tools available to identify DMRs from WGBS data which utilise various algorithms. The use and comparison of three DMR callers resulted in a common set of DMRs but also unique sets of DMRs depending on the caller [33, 34]. Therefore, we took a conservative approach to identify consensus DMRs determined by the three DMR callers [34–36] and used commonly identified DMRs for downstream analysis.

**Fig. 3:**
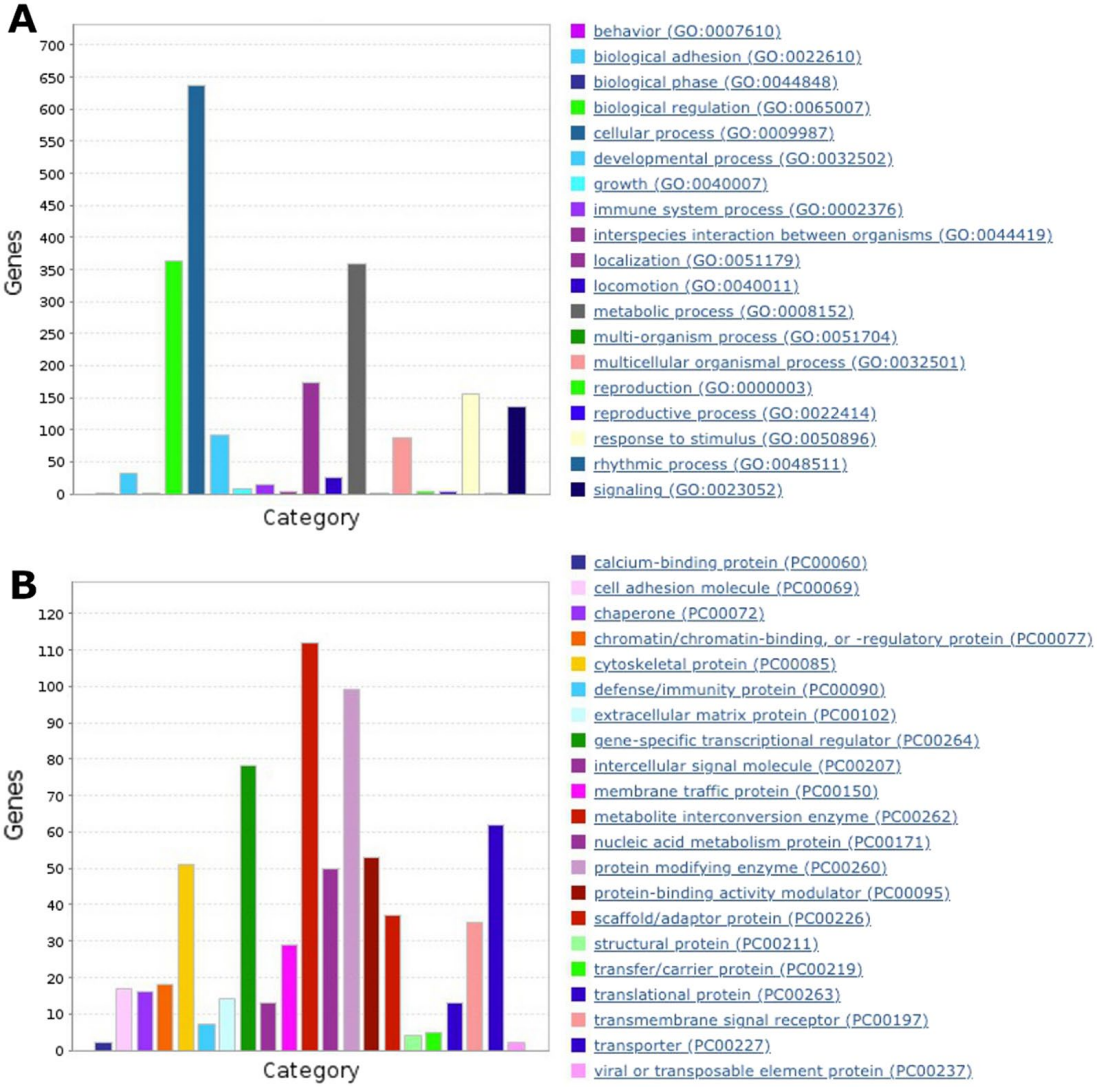
Functional annotation of genomic features corresponding to DMRs. **A** Number of genes (*y*-axis) with GO-slim biological process terms. **B** Number of genes associated with the PANTHER (http://www.pantherdb.org/) protein class categories

Each DMR caller identified between 16 to 19K DMRs between ICSI- and mICSI-derived blastocysts and 2197 of them were commonly identified by all three callers, representing 0.03% of the *Sus scrofa* genome (Table 1 and Additional file 1: Fig. S2). Under-methylated DMRs were more abundant (1621) than over-methylated DMRs (576) in mICSI-derived blastocysts (Additional file 2: Table S2). Functional annotation of the genomic features corresponding to DMRs revealed that DMRs located in the genes involved in cellular process (GO:0009987), biological regulation (GO:0065007) and metabolic process (GO:0008152) were most abundant (Fig. 3). Gene ontology (GO) enrichment analysis revealed that the biological process categories: positive regulation of cell growth (GO:0030307); regulation of Ras protein signal transduction (GO:0046578); and small GTPase mediated signal transduction (GO:0007264) (Additional file 2: Table S3); and the molecular function categories: solute:cation antiporter activity (GO:0015298); cadherin binding (GO:0045296); and methylation-dependent protein binding (GO:0140034) (Additional file 2: Table S4) were amongst the highest enriched GO terms in DMR annotated genes.

**Table 1:** Summary statistics for DMRs identified by three DMR callers

DMR caller^a^	MK	DSS	SQM	All 3^b^
No of DMR	19,474	17,330	16,354	2197
Total length (bp)^c^	9,737,000	5,325,931	49,018,635	813,179
DMR% in the genome^d^	0.40	0.22	2.01	0.03

^a^DMR callers used. MK, MethylKit; DSS; SQM, SeqMonq

^b^DMRs commonly identified by three DMR callers

^c^Total length of DMRs identified

^d^Percentage of DMR sequences in *Sus scrofa* genome

Given that DNA methylation is a dynamic process [29, 30] following fertilisation and during preimplantation embryo development, we also performed a longitudinal comparison to highlight the changes in methylation as the oocyte progressed to the blastocyst stage with and without extra mtDNA. First of all, we identified DMRs between oocyte and ICSI-derived blastocysts indicative of the baseline demethylation changes. Then, these DMRs were compared with the DMRs identified between oocytes and mICSI-derived blastocysts. More than 95% of the DMRs (> 346K) were common to the two DMR data sets, whilst 13–15K DMRs were uniquely represented in each group (Additional file 1: Fig. S5). Using GO enrichment analysis, we identified biological processes over-represented in DMRs unique to each comparison (Additional file 2: Tables S5 and S6). For example, there was positive regulation of ATP metabolic processes (GO:1903580); long-term synaptic potentiation (GO:0060291); and regulation of carbohydrate catabolic processes (GO:0043470). These were most highly enriched (>three-fold) in the oocyte-ICSI blastocyst comparison (Additional file 2: Table S5). On the other hand, GO terms associated with neural retina development, for example retina layer formation (GO:0010842); retina morphogenesis in camera-type eye (GO:0060042); and neural retina development (GO:0003407) were most highly enriched (> three-fold) in the oocyte–mICSI blastocyst comparison (Additional file 2: Table S6).

### Conserved DNA methylation patterns in imprinted genes

**Fig. 4:**
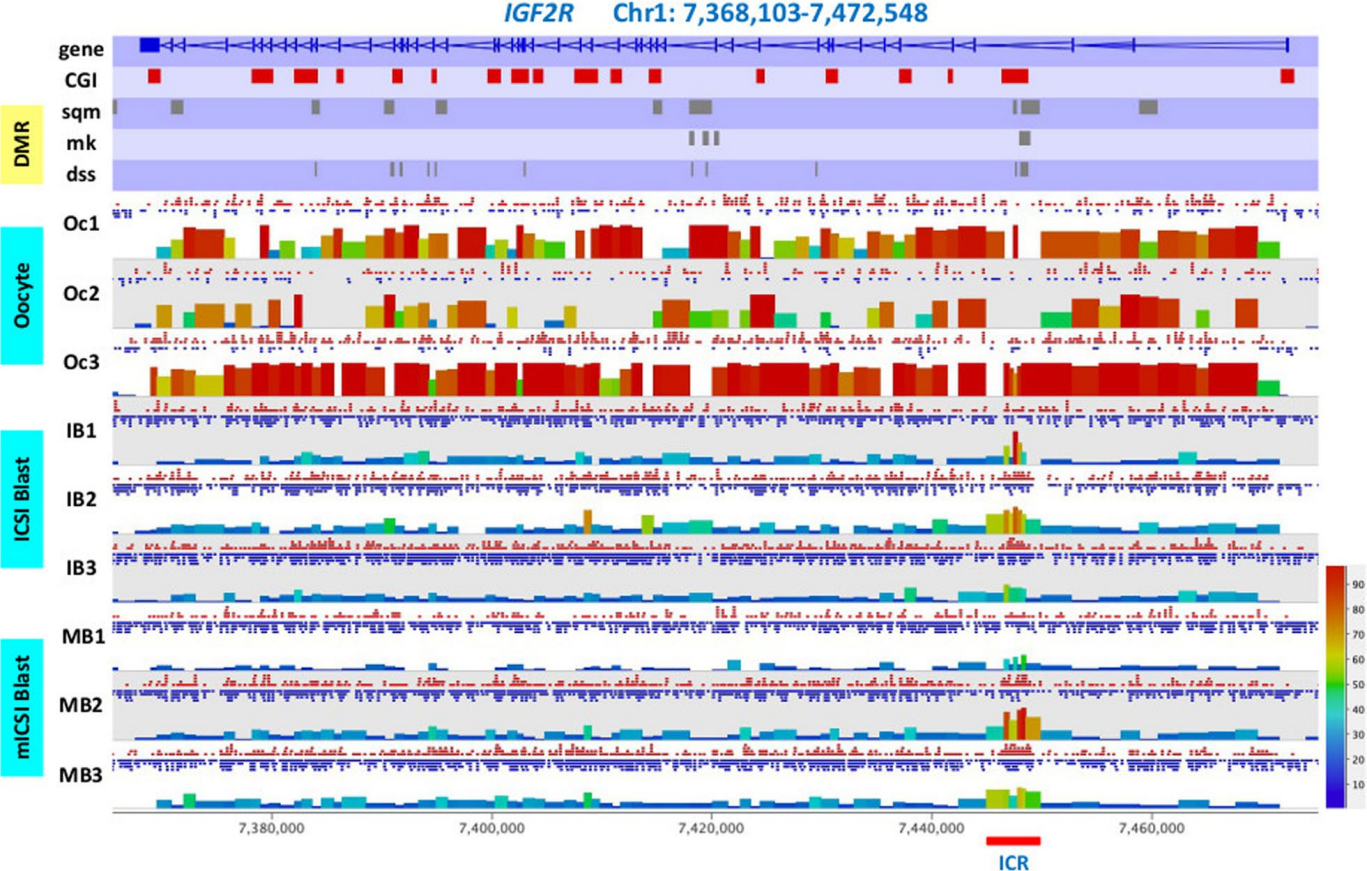
Methylation status of the imprinted gene *IGF2R* in *Sus scrofa* oocytes (Oc) and ICSI-derived (IB) and mICSI-derived (MB) blastocysts. Levels of CpG methylation were calculated using the 100-CpG probe method and displayed as bar histograms in SeqMonk. Red and blue dots above each histogram indicate methylated and unmethylated C counts, respectively. Triplicate BS-seq data for each sample type are shown. Gene and CGI annotated regions are shown at the top panel. DMRs between ICSI- and mICSI-derived blastocysts from the WGBS data were determined by three DMR callers, SeqMonk (sqm), methylKit (mk) and DSS (dss), and indicated by grey boxes under the genomic features at the top part of the panel. The genomic region corresponding to the imprint control region (ICR) identified in human and mouse is indicated by the red bar at the bottom of the panel

Genomic imprinting, parental-specific gene expression in diploid cells, is essential for normal foetal growth and development in mammals [37, 38], and 55 imprinted genes have been reported in *Sus scrofa* [39]. In some cases, parental allele-specific expression is controlled by differentially methylated paternal and maternal alleles, known as imprint control regions (ICR) [40–42]. We identified four imprinted genes from the DMR list (Additional file 2: Table S2) and checked if any of the imprinted gene DMRs were associated with ICRs. Insulin-like growth factor 2 receptor (*IGF2R*) is one of the most characterised imprinted genes. It is involved in multiple biological functions, for example intracellular lysosomal enzyme trafficking, the activation of growth factors/cytokines and IGF2 signalling [43]. The ICR in mouse *IGF2R* is differentially methylated between paternal and maternal alleles. This results in the expression of *IGF2R* from the maternal allele and long non-coding RNA (lncRNA) expression from the paternal allele, which represses the regulatory function of *IGF2R* transcription from the paternal allele [44, 45]. In *Sus scrofa* blastocysts, overall levels of DNA methylation in the *IGF2R* gene body were within normal range (10 to 30%), whilst the ICR corresponding region in the 3rd intron of *IGF2R,* Chr1: 7,444,959–7,449,920, showed elevated levels of methylation (40 to 70%) (Fig. 4). Although it was not possible to determine parental allele-specific DNA methylation patterns in this study, this could represent the signature for the ICR in *Sus scrofa IGF2R*. Two overlapping DMRs were identified in the region of the ICR between nt: 7,448,174 and 7,448,750, which exhibited higher levels of DNA methylation in mICSI-derived blastocysts (Fig. 4 and Additional file 2: Table S2) and represents 1/10 of the length of the putative ICR. We also assessed other putative ICRs in *KCNQ1*, *GNAS* and *MEST* [41, 42, 46]. Similarly, higher levels of methylation were found in these putative ICRs than in other regions of their gene bodies, but no common DMRs were found in these ICRs (Additional file 1: Fig. S6). Several DMRs were found in other genic regions of *KCNQ1*. Overall, ICSI- and mICSI-derived blastocysts did not show major differences in methylation levels at putative ICRs of imprinted genes.

### Differential expression of genes between ICSI- and mICSI-derived blastocysts

In order to determine if there was any association between the DMRs and gene expression, we performed transcriptome analysis of ICSI- and mICSI-derived blastocysts through RNAseq. Single blastocysts underwent RNA extraction and were sequenced using the Illumina NovaSeq platform with five replicates for each type (Additional file 2: Table S7). Over 38 million reads per blastocyst had genomic features assigned and were used for downstream analyses. PCA identified a minor but significant batch effect from the library preparation procedure, therefore, this was factored as a covariate in the linear model. After removal of the batch effect, ICSI- and mICSI-derived libraries formed two distinctive groups, as indicated by PCA plot (Additional file 1: Fig. S7) suggesting distinct gene expression differences between the two types of blastocysts. To increase data reliability, genes with low levels of expression were filtered out (see Methods) for downstream analysis. In all, 10,542 and 10,518 genes had at least 1 count per million reads (CPM) after TMM normalisation and correction for batch effect in ICSI- and mICSI-derived blastocyst RNAseq data, respectively (Additional file 2: Table S7). This indicates the number of genes expressed in blastocysts and is comparable to other reports [28, 47].

In all, differential gene expression analysis identified 52 genes, of which 24 were upregulated and 28 were downregulated in mICSI-derived blastocysts when compared with ICSI-derived blastocysts (Additional file 1: Figs. S8 and S9A and Table 2). These DEGs were statistically significant after employing the FDR (false discovery rate) test (*p.adj* < 0.05) for all RNAseq data sets. Indeed, this represents a significant increase in the number of DEGs identified between ICSI- and mICSI-derived blastocysts under strict statistical conditions when compared with a previous report that employed a porcine-specific embryo array [16]. Amongst these DEGs, we did not find any imprinted genes indicating that the DMRs identified in *IGF2R* and *KCNQ1* did not appear to affect their expression at a significant level (Additional file 1: Fig. S10). We also examined genes catalysing cytosine methylation and demethylation. *TET1* and *TET2*, which have functions in DNA demethylation [48, 49], showed higher levels of expression in blastocysts, whilst the three DNA methyltransferase (*DNMT*) genes [50] were moderately expressed, as previously reported (Additional file 1: Fig. S9B) [28]. Since mitochondrial supplementation induced modulation of *POLG* DNA methylation and increased mtDNA replication prior to embryonic genome activation [16, 19], we investigated the expression of a subset of genes involved in EGA, as listed in [28]. There were variations amongst the RNAseq samples, but no significant differences or obvious trends were identified (Additional file 1: Fig. S9C).

**Table 2:** Differentially expressed genes between ICSI- and mICSI-derived blastocysts

Gene_id^a^	Chr/Scafold^b^	Start	End	Strand	Gene_symbol	Gene_biotype	Description	logFC^c^	adj.P.Val^d^
*Upregulated in mICSI-derived blastocysts relative to ICSI*									
ENSSSCG00000050793	9	47,00,784	47,00,878	−	ssc-mir-10390	miRNA	ssc-mir-10390 [Source:miRBase;Acc:MI0033404]	8.41	1.46E−08
ENSSSCG00000030088	AEMK02000569.1	7,21,734	7,43,707	+		protein_coding	colony stimulating factor 2 receptor alpha subunit [Source:NCBI gene;Acc:100620339]	6.16	4.90E−02
ENSSSCG00000045022	14	7,38,21,782	7,38,28,231	+		lncRNA	NULL	6.02	1.30E−02
ENSSSCG00000045442	AEMK02000694.1	25,505	52,916	−		protein_coding	leucine carboxyl methyltransferase 1-like [Source:NCBI gene;Acc:100625764]	6.00	1.30E−02
ENSSSCG00000021584	15	12,09,85,245	12,09,91,318	+	CDK5R2	protein_coding	cyclin dependent kinase 5 regulatory subunit 2 [Source:HGNC Symbol;Acc:HGNC:1776]	5.67	2.94E−02
ENSSSCG00000047552	9	7,63,60,171	7,63,60,887	+		protein_coding	NULL	5.40	2.94E−02
ENSSSCG00000016816	16	1,95,47,009	1,99,20,515	−	ADAMTS12	protein_coding	ADAM metallopeptidase with thrombospondin type 1 motif 12 [Source:HGNC Symbol;Acc:HGNC:14605]	5.16	2.94E−02
ENSSSCG00000045298	9	7,73,07,796	7,73,15,488	−		lncRNA	NULL	4.80	4.41E−02
ENSSSCG00000051325	1	24,83,28,317	24,84,48,089	−		lncRNA	NULL	4.31	4.96E−02
ENSSSCG00000010017	14	4,78,79,317	4,79,02,699	+	SMTN	protein_coding	smoothelin [Source:NCBI gene;Acc:414369]	3.56	1.41E−03
ENSSSCG00000043834	AEMK02000692.1	81,135	86,443	+		protein_coding	NULL	3.46	3.82E−03
ENSSSCG00000017592	12	2,74,50,805	2,75,26,985	−	MBTD1	protein_coding	mbt domain containing 1 [Source:HGNC Symbol;Acc:HGNC:19866]	2.93	3.72E−03
ENSSSCG00000015729	15	2,96,75,820	2,97,14,079	−	TSN	protein_coding	translin [Source:HGNC Symbol;Acc:HGNC:12379]	2.68	7.98E−04
ENSSSCG00000046732	AEMK02000589.1	1,03,902	1,10,514	+		lncRNA	NULL	2.65	3.45E−02
ENSSSCG00000050138	AEMK02000256.1	1,71,630	1,81,191	+		lncRNA	NULL	2.49	1.25E−03
ENSSSCG00000014362	2	14,21,02,456	14,21,15,344	−	HBEGF	protein_coding	heparin binding EGF-like growth factor [Source:HGNC Symbol;Acc:HGNC:3059]	2.29	1.49E−02
ENSSSCG00000031712	5	6,27,42,315	6,27,59,190	+	MFAP5	protein_coding	microfibril associated protein 5 [Source:HGNC Symbol;Acc:HGNC:29673]	2.06	2.94E−02
ENSSSCG00000007043	17	1,44,20,656	1,44,76,189	−	GPCPD1	protein_coding	glycerophosphocholine phosphodiesterase 1 [Source:HGNC Symbol;Acc:HGNC:26957]	1.79	4.18E−02
ENSSSCG00000015604	9	13,15,47,084	13,15,60,527	+	NEK2	protein_coding	NIMA related kinase 2 [Source:HGNC Symbol;Acc:HGNC:7745]	1.57	4.90E−02
ENSSSCG00000011704	13	8,97,42,893	8,98,89,187	−	WWTR1	protein_coding	WW domain containing transcription regulator 1 [Source:HGNC Symbol;Acc:HGNC:24042]	1.33	3.29E−02
ENSSSCG00000015144	9	5,02,58,971	5,05,09,799	+	GRAMD1B	protein_coding	GRAM domain containing 1B [Source:HGNC Symbol;Acc:HGNC:29214]	1.28	2.55E−02
ENSSSCG00000020927	13	10,95,87,256	10,96,23,787	−	SLC2A2	protein_coding	solute carrier family 2 member 2 [Source:NCBI gene;Acc:397429]	1.26	9.43E−03
ENSSSCG00000034282	15	11,72,44,763	11,74,51,261	−	ABCA12	protein_coding	ATP binding cassette subfamily A member 12 [Source:HGNC Symbol;Acc:HGNC:14637]	1.09	4.96E−02
ENSSSCG00000030211	12	1,97,55,737	1,97,88,952	−	NBR1	protein_coding	NBR1 autophagy cargo receptor [Source:HGNC Symbol;Acc:HGNC:6746]	1.07	1.67E−02
*Downregulated in mICSI-derived blastocysts relative to ICSI*									
ENSSSCG00000018062	MT	2206	2273	+		Mt_tRNA	product = tRNA-Val	− 11.31	4.32E−06
ENSSSCG00000018070	MT	6129	6196	+		Mt_tRNA	product = tRNA-Trp	− 8.93	1.34E−04
ENSSSCG00000018079	MT	8891	8957	+		Mt_tRNA	product = tRNA-Lys	− 8.81	2.02E−05
ENSSSCG00000018073	MT	6379	6444	−		Mt_tRNA	product = tRNA-Cys	− 8.30	1.55E−04
ENSSSCG00000018071	MT	6203	6270	−		Mt_tRNA	product = tRNA-Ala	− 7.55	8.92E−04
ENSSSCG00000032749	8	8,93,21,491	8,93,35,395	+	PCDH18	protein_coding	protocadherin 18 [Source:HGNC Symbol;Acc:HGNC:14268]	− 6.48	9.43E−03
ENSSSCG00000018072	MT	6272	6346	−		Mt_tRNA	product = tRNA-Asn	− 6.10	6.78E−03
ENSSSCG00000012257	X	3,89,29,231	3,90,06,222	+		protein_coding	monoamine oxidase A [Source:NCBI gene;Acc:414424]	− 5.94	3.09E−02
ENSSSCG00000051497	5	10,21,87,453	10,27,20,929	−		lncRNA	NULL	− 5.77	7.62E−03
ENSSSCG00000021343	15	74,99,026	76,32,655	+	ZEB2	protein_coding	zinc finger E-box binding homeobox 2 [Source:HGNC Symbol;Acc:HGNC:14881]	− 5.56	3.45E−02
ENSSSCG00000050427	2	12,87,99,599	12,92,30,191	−		lncRNA	NULL	− 5.53	2.38E−02
ENSSSCG00000001097	7	1,95,87,685	1,98,38,093	−		protein_coding	RHO family interacting cell polarization regulator 2 [Source:NCBI gene;Acc:100154661]	− 5.35	2.12E−02
ENSSSCG00000037634	7	6,24,71,228	6,24,79,223	+	FOXA1	protein_coding	forkhead box A1 [Source:HGNC Symbol;Acc:HGNC:5021]	− 5.18	3.45E−02
ENSSSCG00000001484	7	2,64,46,306	2,65,22,965	−	TINAG	protein_coding	tubulointerstitial nephritis antigen [Source:HGNC Symbol;Acc:HGNC:14599]	− 5.14	4.87E−02
ENSSSCG00000045069	7	1,68,85,827	1,68,91,770	−		lncRNA	NULL	− 4.96	4.56E−02
ENSSSCG00000018074	MT	6444	6509	−		Mt_tRNA	product = tRNA-Tyr	− 4.73	2.94E−02
ENSSSCG00000035347	14	10,60,13,495	10,67,13,121	+	CYP2C42	protein_coding	cytochrome P450 C42 [Source:NCBI gene;Acc:403111]	− 3.95	3.31E−03
ENSSSCG00000015780	15	4,50,83,606	4,53,01,158	+	STOX2	protein_coding	storkhead box 2 [Source:HGNC Symbol;Acc:HGNC:25450]	− 3.41	2.94E−02
ENSSSCG00000023204	3	4,13,37,071	4,13,93,997	+	AXIN1	protein_coding	axin 1 [Source:HGNC Symbol;Acc:HGNC:903]	− 3.20	1.35E−03
ENSSSCG00000039416	15	1,56,63,494	1,56,67,233	+	CXCR4	protein_coding	C-X-C motif chemokine receptor 4 [Source:NCBI gene;Acc:396659]	− 2.54	2.55E−02
ENSSSCG00000016925	16	3,72,99,116	3,73,05,116	−	PLK2	protein_coding	polo like kinase 2 [Source:HGNC Symbol;Acc:HGNC:19699]	− 2.38	4.91E−02
ENSSSCG00000022390	X	4,17,26,526	4,17,49,726	+	RGN	protein_coding	regucalcin [Source:HGNC Symbol;Acc:HGNC:9989]	− 1.91	9.43E−03
ENSSSCG00000006562	4	9,57,59,073	9,58,64,458	+	GATAD2B	protein_coding	GATA zinc finger domain containing 2B [Source:HGNC Symbol;Acc:HGNC:30778]	− 1.68	9.43E−03
ENSSSCG00000004390	1	7,50,33,098	7,51,69,987	−	SESN1	protein_coding	sestrin 1 [Source:HGNC Symbol;Acc:HGNC:21595]	− 1.62	1.67E−02
ENSSSCG00000026784	2	7,61,25,926	7,61,46,733	+	LMNB2	protein_coding	lamin B2 [Source:HGNC Symbol;Acc:HGNC:6638]	− 1.55	1.44E−02
ENSSSCG00000014267	2	13,37,08,266	13,39,51,438	−		protein_coding	Rap guanine nucleotide exchange factor 6 [Source:NCBI gene;Acc:100521255]	− 1.54	1.44E−02
ENSSSCG00000047299	AEMK02000489.1	43,232	45,101	+	RN18S	rRNA	18S ribosomal RNA [Source:NCBI gene;Acc:100861538]	− 0.71	3.89E−06
ENSSSCG00000012847	2	4,35,125	4,73,744	+	TALDO1	protein_coding	transaldolase 1 [Source:NCBI gene;Acc:100514210]	0.85	4.90E−02

^a^Ensembl (https://m.ensembl.org/index.html) gene ID

^b^Chromosome or scaffold number where gene is located

^c^Log 2-fold change relative to ICSI-derived blastocyst

^d^Adjusted *p*-value

**Fig. 5:**
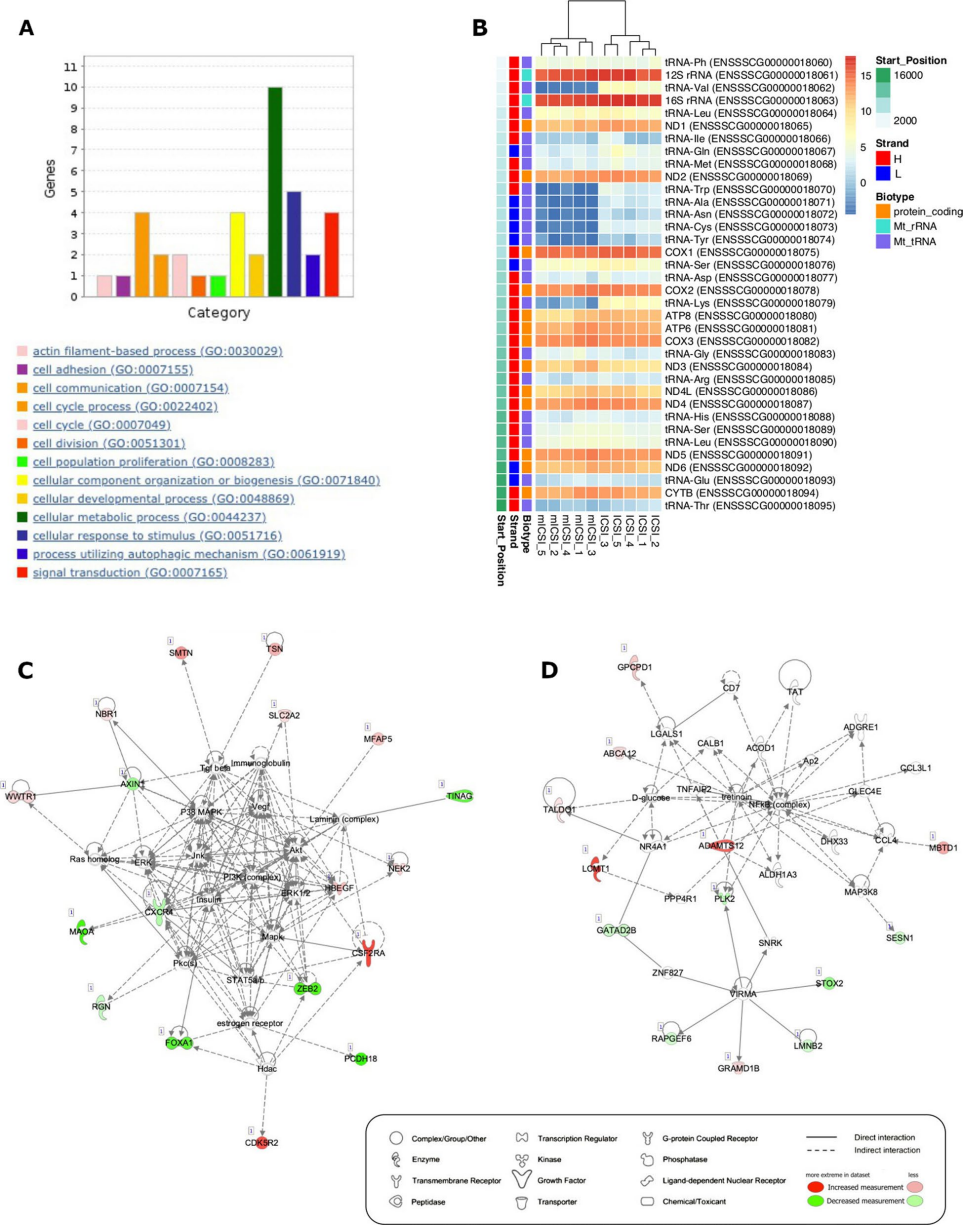
Analysis of differentially expressed genes between *Sus scrofa* ICSI- and mICSI-derived blastocysts. **A** Functional annotations of DEGs in the biological process subcategory cellular process (GO:0009987). **B** Heatmap displaying mtDNA-encoded genes in *Sus scrofa* ICSI- and mICSI-derived blastocysts. Rows and columns show individual genes and RNAseq samples, respectively, ordered by position in the *Sus scrofa* mtDNA sequence. Each tile in the main matrix represents the levels of expression of a single gene in a single RNAseq data set. Colour of tile indicates levels of expression, and a scale is presented on the right. The DNA strand of encoded genes, biotype of genes, and start position in mtDNA are also presented in colour tiles on the left. **C** and **D** Analysis of signalling pathways for differentially expressed genes using IPA. Identified signalling network 1 (**C**) and network 2 (**D**) are shown. Types of molecules are drawn in different shapes and genes that are up- or down-regulated in mICSI-derived blastocysts are indicated in red and green, respectively

Out of 52 DEGs, 32 genes had annotation and gene ontology (GO) information, however, this did not point to any GO terms showing significant over-representation, possibly due to the relatively small number of GO terms used for analysis. Amongst the GO terms from these 32 genes, 16 genes had annotations associated with cellular processes (GO:0009987) and 10 were involved in cellular metabolic processes (GO:0044237 in Fig. 5A). MicroRNA *ssc-mir-10390* (ENSSSCG00000050793) showed the highest difference (> 340-fold) amongst the DEGs that were upregulated in mICSI-derived blastocysts (Table 2 and Additional file 1: Fig. S10). Since miRNA *ssc-mir-10390* has not been functionally annotated, we searched miRNA target genes in the *Sus scrofa* genome. We identified 538 potential microRNA target sites located in 166 genes and 96 genes were expressed in blastocysts (Additional file 2: Table S8). Although no putative miRNA target genes were identified in the list of DEGs (Table 2), they could have been silenced by translational repression and/or mRNA degradation mechanisms [51, 52].

To maximise the use of the DEG list and associated expression data to determine if there could be functional implications resulting from the DEG profiles between the two sets of blastocysts, we also performed ingenuity pathway analysis [53] to determine if any biological networks were significantly affected by the DEGs. Two biological networks appeared to be affected. As shown for network 1 (Fig. 5C), which involves haematological system development and function, inflammatory response, and tissue morphology, 18 out of 35 genes in this pathway were differentially expressed. For network 2 (Fig. 5D), which associates with cell cycle, drug metabolism, lipid metabolism, 13 out of 35 genes were differentially expressed. These might be linked to the developmental differences previously observed between ICSI- and mICSI-derived blastocysts [16].

One of the most striking aspects associated with the list of DEGs was that seven mtDNA-encoded t-RNAs were downregulated in mICSI-derived blastocysts (Table 2). Both strands of the entire mitochondrial genome are transcribed as long polycistronic transcripts which undergo multiple processing steps before individual RNAs become functional [54]. Amongst the seven tRNAs, three are encoded on the heavy (H)-strand and four on the light (L)-strand (Additional file 2: Table S9 and Fig. 5B). Other mtDNA genes did not show significant differences between ICSI- and mICSI-derived blastocysts suggesting that mitochondrial supplementation affected tRNA processing and/or turnover in blastocysts.

### Association between DMRs and DEGs between ICSI and mICSI blastocysts

**Fig. 6:**
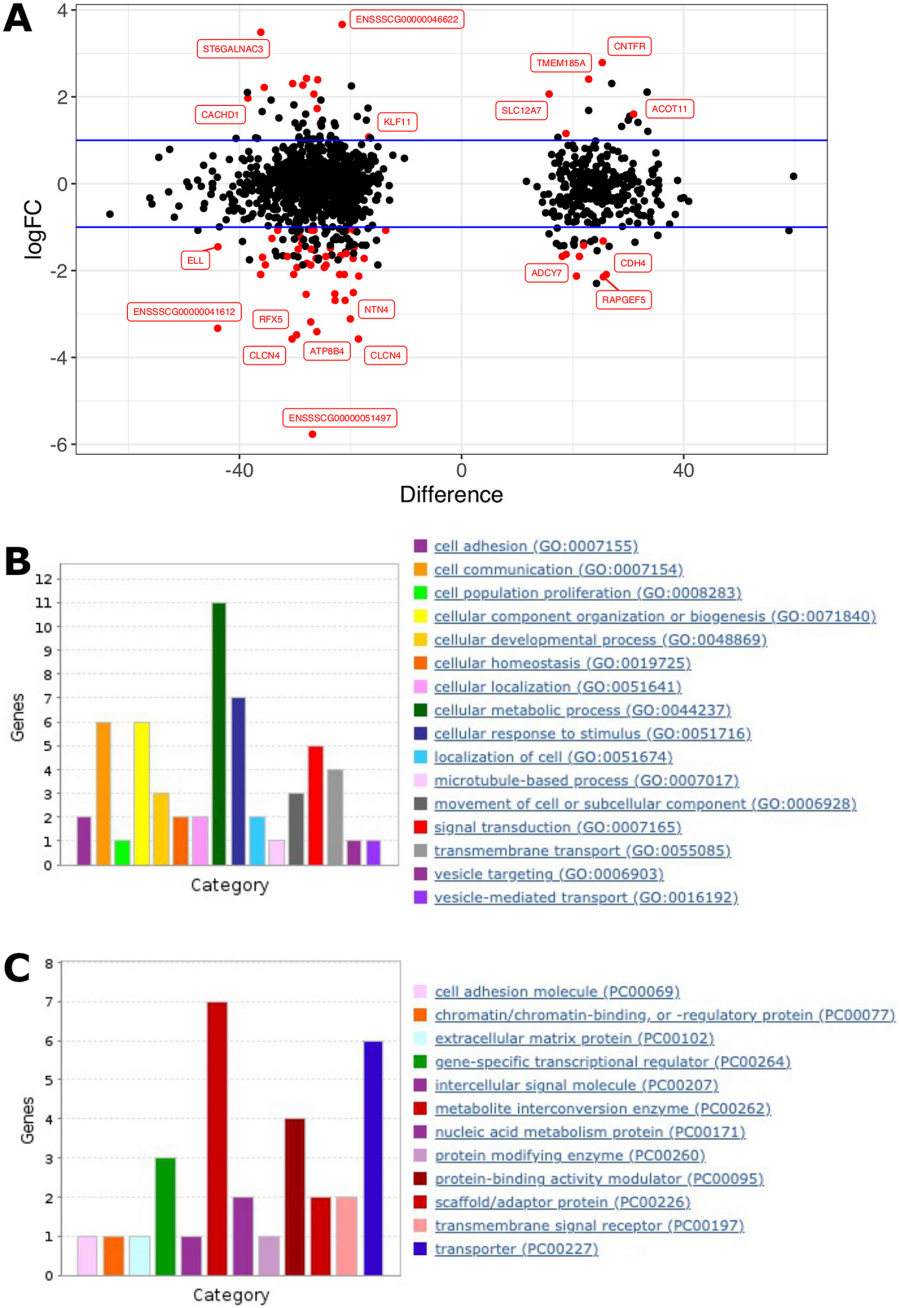
Integration of DMRs with RNAseq results. DMRs associated with possible DEGs were identified through data filtration. **A** Annotated DMRs with > two-fold expression difference (log2 fold change on *y*-axis) and a raw *p*-value (< 0.05) are highlighted in red. Difference in methylation level (mICSI-derived blastocysts—ICSI-derived blastocysts %) is indicated on the *x*-axis. **B** and **C** Functional annotation of genes with both levels of methylation and expression affected in mICSI-derived blastocysts. **B** Number of genes (*y*-axis) with GO biological process terms in the cellular process (GO:0009987) category. **C** Number of genes associated with the PANTHER (http://www.pantherdb.org/) protein class categories

Since we had found 2197 DMRs commonly identified by the three callers (Table 1), we investigated if there was an association with any of the DEGs. Based on the annotation of the DMR locations (Additional file 2: Table S2), none of the DMRs were linked with significant DEGs as determined by adjusted *p*-value (FDR test; Table 2). When we integrated the DMR and DEG data sets by using a list of DEGs filtered by fold change (> two-fold) and raw *p*-value (< 0.05), we identified 72 DMRs amongst the 55 unique genes, showing potential associations (Fig. 6A and Additional file 2: Table S10). Almost all of the DMRs were located in the intragenic regions of these genes except for one in the promoter region of a lncRNA (ENSSSCG00000043867). There were seven lncRNAs in the integrated DMR and DEG list (Additional file 2: Table S10) and none of them were annotated. Amongst the remaining 48 protein coding genes, 27 had functional annotations associated with cellular processes (GO:000987) with various subcategories (Fig. 6B). Cellular metabolic processes (GO:0044237) were highest amongst these and associated protein functions such as metabolite interconversion enzyme and transporter were also abundant (Fig. 6C). These are potential genes exhibiting altered levels of DNA methylation and gene expression as a result of mitochondrial supplementation that would influence metabolic processes in blastocysts.

## Discussion

Mitochondrial supplementation of mtDNA-deficient oocytes has the potential to improve fertilisation outcomes and embryo development [16]. To this extent, we have previously demonstrated that mitochondrial supplementation modulated gene expression profiles in *Sus scrofa* embryos up to the blastocyst stage and DNA methylation of the nuclear encoded mtDNA-specific replication factor, *POLG*, resulting in appropriate levels of mtDNA copy number being established by the blastocyst stage [16, 19]. This suggests that mitochondrial supplementation induces changes in the gene expression profiles of the nuclear genome by altering the patterns of epigenetic programming established through the waves of DNA de/methylation that take place throughout oogenesis, namely as the primordial germ cell differentiates into a mature metaphase II oocyte. In this study, we have taken a global approach to assessing the impact of mitochondrial supplementation on the nuclear genome. In this respect, we have shown that 52 genes were differentially expressed and over 2000 local genomic regions were differentially methylated in *Sus scrofa* blastocysts as a result of adding ~ 800 extra copies of mtDNA into in vitro matured sister oocytes. We have previously reported the association between mtDNA copy number and nuclear DNA methylation profiles in tumour cells [55, 56]. Our results provided further evidence that a small change (< 1%) in mtDNA copy number could affect the epigenetic profile of the nuclear genome. This also suggests that the synchrony established between the two genomes, namely genomic balance [11, 21], during oogenesis is altered and may have consequences for post-fertilisation epigenetic programming following the fusion of the paternal and maternal genomes and activation of the newly formed genome.

Our comparison of ICSI- and mICSI-derived blastocysts through WGBS analysis revealed that mitochondrial supplementation did not alter DNA methylation patterns at a global chromosome level (Additional file 1: Fig. S4). Nevertheless, we observed slightly higher levels of DNA methylation in ICSI-derived blastocysts (Additional file 2: Table S1 and Fig. 1). Although none of the genes catalysing cytosine methylation and demethylation were significantly different in their levels of expression (Additional file 1: Fig. S9), there were minor differences, such as observed for *APOBEC1* (Additional file 1: Fig. S10), which deaminates 5 hmC to 5 hmU [48, 57], and could, thus, contribute to the slightly higher overall methylation status in ICSI-derived blastocysts.

Our conservative approach to the analysis of DMRs, namely using DMRs identified by three methylation-specific callers, revealed that the main effect of mitochondrial supplementation on the DNA methylation of the nuclear genome occurred in local genomic regions (Table 1 and Additional file 2: Table S2). To this extent, the majority of DMRs were found in intragenic regions of genes involved in biological regulation (GO:0065007), cellular processes (GO:0009987) and metabolic processes (GO:0008152) (Fig. 3), some of which could influence transcription of critical factors for development and growth [58]. We have also demonstrated the presence of uniquely methylated genomic regions between ICSI- and mICSI-derived blastocysts by longitudinal comparison of DMRs, capturing differences in the epigenetic reprograming processes (Additional file 1: Fig. S5). Using this approach, we showed potential differences in the processes of DNA demethylation associated with mitochondrial supplementation and the resultant link with biological relevance (Additional file 2: Tables S5 and S6). Although these DMRs may or may not directly change transcriptional regulation in blastocysts, they could be responsible for downstream developmental stage-dependent and tissue-specific effects with the potential to also induce transgenerational effects [59, 60].

A key facet of early developmental programming is genomic imprinting. Given that DNA methylation is established in the maternal and paternal germlines, these imprinted DNA methylation patterns are maintained in the epigenetic reprograming process that takes place during early embryogenesis [37, 59]. However, it appears that mitochondrial supplementation does not affect the putative imprinting control regions [37, 40] of *Sus scrofa* imprinted genes (Figs. 4 and Additional file 1: Fig. S6). To this extent, we did not find DMRs in any of the putative ICRs we assessed except for a portion of the ICR in *IGF2R*. Nevertheless, none of the known imprinted genes [39] and genes involved in embryonic genome activation [28, 61] showed differences in gene expression (Additional file 1: Fig. S9) suggesting that mitochondrial supplementation does not adversely affect imprinting patterns in the preimplantation embryo [16].

We have previously shown that seven genes were differentially expressed in blastocysts derived from mtDNA-deficient oocytes fertilised by mICSI and non-mtDNA-deficient oocytes fertilised through ICSI using data from microarray analysis after applying FDR [16]. In this study, we identified 52 DEGs in mICSI-derived blastocysts compared with ICSI-derived blastocysts by RNAseq (Table 2 and Additional file 1: Fig. S9A). We used a higher number of RNAseq biological replicates (*n* = 5) and the same quality oocytes for both mICSI and ICSI, which allowed us to directly address the impact of solely adding extra copies of mtDNA whilst the previous analysis addressed the effect of mitochondrial supplementation on mtDNA-deficient oocytes at the blastocyst stage [16]. We found genes involved in cellular metabolic process (GO:0044237) to be most abundant amongst the DEGs (Fig. 5A). This is consistent with the findings associated with introducing extra copies of mtDNA into mtDNA-deficient oocytes [19]. This suggests that extra copies of mtDNA could promote cellular metabolism just after fertilisation. From studies in other cellular systems, it is evident that alterations to metabolic programming can produce by-products that help mediate transition from a methylated to demethylated state [62] and may account for the slightly lower levels of methylation observed in mISCI-derived blastocysts.

The key gene networks affected by mitochondrial supplementation included haematological system development and function, inflammatory response, tissue morphology, cell cycle, drug metabolism and lipid metabolism (Fig. 5C and D). Previously, we observed a defect in the cardiac structure in first- and second-generation mICSI-derived murine offspring [20]. There are two genes, which have potential functions in heart development, found in the identified functional gene network 1 (Fig. 5C). Regucalcin (RGN), which has been shown to increase rat heart sarcoplasmic reticulum Ca^2+^-ATPase activity and ATP‐dependent Ca^2+^ uptake, is a key molecule in heart muscle cell regulation through Ca^2+^ signalling, and has been suggested to play a pathophysiological role in heart failure [63, 64]. On the other hand, Heparin-binding EGF-like growth factor (HBEGF) is involved in cardiac valve development, as demonstrated in mice [65]. The observed influence of mitochondrial supplementation on haematological system development and tissue morphology gene network in *Sus scrofa* and defective cardiac structure in mICSI-derived murine offspring are concerns if mitochondrial supplementation were to be introduced into clinical practice.

Amongst the DEGs in the mICSI-derived blastocyst cohort, there were two candidate genes with the potential to have an impact on development and subsequent methylation patterns. The first is microRNA *ssc-mir-10390*, which was over 340-fold upregulated in mICSI-derived blastocysts (Table 2). We identified 538 potential microRNA target sites in the *Sus scrofa* genome in 166 genes and 96 of these genes were expressed in blastocysts (Additional file 2: Table S8). miRNA *ssc-mir-10390* target genes could be silenced by translational repression and/or mRNA degradation [51, 52], which would have a significant impact on growth and development. The second gene is *FOXA1*, known as the 'pioneer transcription factor', which has an ability to open condensed chromatin, allowing transcriptional enhancers access to initiate transcription [66]. It has also been shown that the genomic regions surrounding FOXA1-binding sites were hypomethylated inducing DNA demethylation around binding sites. Consequently, the observed difference in *FOXA1* expression between ICSI- and mICSI-derived blastocysts could be associated with the DMRs we identified. 

One of the most striking results from the DEG analysis was a subset of mitochondrial tRNAs that were significantly under-regulated (25- to 2500-fold lower) following mitochondrial supplementation (Table 2). This could significantly affect protein synthesis of the genes associated with the mitochondrial genome and potentially have a huge impact on the function of the electron transport chain [67]. In this respect, RGN is also localised in the mitochondrion as well as the cytoplasm, microsomes and nucleus, and has an inhibitory effect on aminoacyl tRNA synthetase [64]. We observed downregulation of RGN in mICSI-derived blastocysts, which might lead to increased activity of aminoacyl tRNA synthetase and disrupt the balance of the tRNA molecule number and turnover rate. Each of the two mtDNA strands is transcribed as a single polycistronic transcript and processed [54], thus, generating one copy of each encoded mRNA, rRNA and tRNA. However, it remains to be determined if there are any selective transcription mechanisms for mitochondrial tRNA genes to ensure a high tRNA-to-mRNA ratio; how mitochondria accumulate enough tRNAs for translation; and if the turnover rate for mitochondrial tRNAs is slower than mtDNA-encoded mRNAs [68]. Why only seven out of the 22 mitochondrial tRNAs were selectively downregulated, and other tRNAs and protein coding genes were unchanged, are interesting questions for fundamental mitochondrial biogenesis.

The integration of DMRs and transcriptomic data identified candidate genes with potential associations with changes in the methylation status and levels of gene expression (Fig. 6 and Additional file 2: Table S10). In all, 55 genes exhibited differences in gene expression between mICSI- and ICSI-derived blastocysts with DMRs in intragenic regions. Again, cellular metabolic process (GO:0044237) was the most abundant functional annotation category (Fig. 6B), consistent with the result of the other DEG list (Fig. 5A). Four genes (*SHANK2*, *CMIP*, *CDH4* and *NHS*) have multiple DMRs located in their gene bodies and were downregulated in mICSI-derived blastocysts (Additional file 1: Fig. S10). Amongst the integrated DMR and DEG list (Additional file 2: Table S10) and the DEG list (Table 2), we found 15 lncRNA. Although none of these have functional annotation, recent accumulated evidence suggests that lncRNAs have important regulatory roles in chromatin architecture, chromatin remodelling, transcriptional regulation, and other associated functions [69, 70]. For example, *CCAT1-L* lncRNA is transcribed upstream of human *MYC*, which modulates intrachromatin loops between enhancers and promoters by facilitating the formation of enhancer–promoter loops at the *MYC* locus [71]. We identified that the lncRNA (ENSSSCG00000045298) located at chromosome 9: 77,307,796–77,315,488 is upregulated in mICSI-derived blastocysts. We found a novel protein encoding gene (ENSSSCG00000047552) located in close proximity at the same locus was also upregulated (Table 2). Interestingly, this is the *PEG10*-imprinted gene cluster locus, containing several other imprinted genes (*SGCE*, *CASD1*, *PPP1R9A*, *ASB4* and *PON2*) at the locus [39]. *PEG10* is downregulated 2.8-fold and *COL28A1* at the same locus is upregulated 5.7-fold (raw *p*-value < 0.05) (Additional file 1: Fig. S10). It is not known whether this is coincidental or directly associated with lncRNA expression. However, expression of the lncRNA *Airn* in the *IGF2R* locus and *Kcnq1ot1*/*Lit1*in the *KCNQ1* locus from the paternal allele induces repression of a few genes in the paternal allele at both loci [40, 72, 73]. Therefore, the lncRNAs we identified may have significant roles in transcriptional regulation of associated neighbouring genes.

## Conclusions

We have demonstrated that the addition of just an extra ~ 800 copies of mtDNA into in vitro matured sister oocytes (i.e. autologous mtDNA supplementation) can have a significant impact on both gene expression and DNA methylation profiles in *Sus scrofa* blastocysts. Some changes in the DNA methylation status and gene expression at an early stage may have an effect on specific tissue-types or later in life. The effect could be amplified as the blastocyst develops into an embryo proper, a foetus, and ultimately an offspring. Alternatively, these DMRs and DEGs may not necessarily have an impact as differences at the blastocyst stage may be corrected and disappear at later stages of development. Indeed, these outcomes would be very important for further investigation to follow mICSI-derived offspring until the adult stage to assess the effect of these molecular changes on growth and development and the long-term effect of mitochondrial supplementation before considering human clinical practice.

## Materials and methods

### Cumulus–oocyte complexes collection and in vitro maturation (IVM)

Pairs of gilt ovaries were collected from a local abattoir and kept as individual pairs prior to and during transport to the laboratory in warm 0.9% NaCl solution. The cumulus–oocyte complexes (COCs) from each ovary pair were aspirated from follicles with diameters of 3–6 mm using an 18 G needle. The COCs from each ovary pair were then washed 3 times in handling media (25 mM Hepes–TCM199, Gibco®) supplemented with 10% sow follicular fluid (SFF) and cultured with COCs from their respective ovary pair for 42—44 h in 500 µl pre-equilibrated in vitro maturation media (TCM199 media supplemented with 0.80 mM Na-pyruvate, 0.61 mM l-glutamine, 0.88 M cysteamine, 5 µg/ml insulin, 10 IU/ml PMSG, 10 IU/ml HCG, and 0.10 µg/ml EGF and 10% SFF) in a humidified incubator at 38.5 °C with 5% CO_2_ in air.

### Metaphase II (MII) oocyte collection

To collect MII oocytes from each ovary pair, expanded COCs following IVM were briefly treated with 0.1% (0.5mg/ml) of hyaluronidase in the maturation wells with pipetting. The oocytes were then transferred to an individual dish for each ovary pair, and denuded oocytes were washed with a narrow glass pipette to completely remove all cumulus cells. The MII oocytes, which exhibited the presence of the first polar body, were collected into individual 0.2 ml tubes for each ovary pair and used for DNA methylation analysis; or they were subsequently fertilised with or without mitochondrial supplementation.

### Generation of ICSI- and mICSI-derived blastocysts

Expanded blastocyst stage *Sus scrofa* embryos were generated from in vitro matured oocytes by intracytoplasmic sperm injection (ICSI); and autologous mitochondrial supplementation in combination with ICSI (mICSI) using sister oocytes as the source of mitochondrial isolate, as previously described [16]; and as illustrated in Additional file 1: Fig. S1. The presence of mtDNA in the mitochondrial isolate was verified by PCR [17] using DNA extracted from some of the mitochondrial isolate employed for each round of supplementation. Blastocyst development rates for this study are shown in Additional file 2: Table S11. There were no significant differences for survival and embryo development rates between the ICSI and mICSI groups, as analysed by *χ*^2^ test. Single or a pool of six embryos were stored in 0.2 ml PCR tubes in a − 80 °C freezer prior to RNA and DNA extraction, respectively.

### Preparation of WGBS libraries

Total DNA was extracted from pools of 40 to 63 MII oocytes or a pool of six blastocysts by using the QIAamp DNA Micro Kit (QIAGEN, VIC, Australia), according to the manufacturer’s instructions. DNA was eluted in 20 μl of elution buffer. Triplicate DNA samples for oocytes, and ICSI- and mICSI-derived blastocysts were used for WGBS library construction. Preparation of WGBS libraries and Illumina NGS were performed by the South Australian Genomics Centre (SAHMRI, Adelaide, SA, Australia). Briefly, 10 μl of total DNA solution were used for bisulfite treatment and NGS library construction using the Pico Methyl-Seq Library Prep Kit (ZYMO RESEARCH, CA, USA), according to the manufacturer’s instructions. The Illumina NovaSeq S1 flow cell was used to run WGBS libraries using 100-bp paired-end sequencing chemistry.

### WGBS data analysis

WGBS data were analysed following the procedure described in [74] with minor modifications. Firstly, adaptors and poor-quality reads were cleaned from raw sequences using the TrimGalore program v0.4.2 [75] in the paired-end mode with the default adaptor trimming option and additional 10 bp trimming for both the 5' and 3' ends. The quality filtered and trimmed sequences were mapped to the *Sus scrofa* genome sequence (Sscrofa11.1 Accession No. GCF_000003025.6) using Bismark Package v0.22.3 [26]. The following Bismark options were applied: *–bowtie2*; *-N* 1; *-L* 20; *–non_directional*; *–score_min* L,0,−0.2 for paired-end sequence data. Unmapped paired-end reads were then re-analysed using the single-end read mapping mode. Output bam files for each sample were deduplicated using the Bismark package ‘*deduplicate_bismark*’ function to remove PCR duplicates. Deduplicated mapped reads obtained by single- and paired-end mapping modes were combined. SAMtools [76] were used to handle output bam files and obtain mapped sequence read statistics using utilities: ‘*sort*’, ‘*merge*’, ‘*view*', ‘*index*’, ‘*stats*’, ‘*idxstats*’, and ‘*depth*’. Methylation coverage data for the CpG context were extracted using the '*bismark_methylation_extractor*' function with the following options: *–bedGraph –gzip –cytosine_report*. All cytosine sites with at least one count were kept for further analysis.

Genome-wide methylation coverage data were analysed and visualised using the SeqMonk software package version 1.48.0 [36]. For unbiased methylation analysis, 100-CpG probes were defined using the '*Read Position Probe Generator*' [27] from all WGBS data sets used in this study, which resulted in 528,995 100-CpG probes throughout the *Sus scrofa* genome. The SeqMonk bisulfite quantitation pipeline was used with the condition of 1 minimum count to include a position; and 5 minimum observations to include a feature [28]. Genomic features in the *Sus scrofa* Sscrofa11.1_v100_assembly, e.g. intra- and intergenic regions and CpG island (CGI), were used to calculate methylation levels from the overlapping 100-CpG probe data. Gene promoters were defined as genomic regions of 2500 bp upstream of transcriptional start sites. Gene and CGI density were calculated as a sum of the genomic feature length (bp) in 2 Mbp bins throughout the *Sus scrofa* genome using the *ggplot2* package [77] '*stat_bin*' function.

### DMR analysis

DMRs between ICSI and mICSI data sets were analysed by three DMR callers in order to obtain consensus DMRs. First, a logistic regression test was carried out for 100 CpG probes with a *p*-value cut-off of 0.05 and 10 minimum observations using SeqMonk. Second, the R package *methylKit* [35] was used for DMR identification with the following conditions: 500 bp window size, 250 bp step size, minimum of 1 coverage per CpG site in at least two out of three replicates, minimum of 10 counts per window, minimum methylation difference of 25%, and *q*-value cut-off of 0.01. Third, the Bioconductor package *DSS* [34] was used for DMR analysis with a *p*-value cut-off of 0.01, 20 minimum CpG sites per window and use of the smoothing option. DMRs identified by all three callers (Table 1) were imported into SeqMonk as annotation tracks and overlapping DMRs were filtered and retained as consensus DMRs (Additional file 2: Table S2) for downstream analysis.

### RNA extraction from blastocysts, RNAseq library construction and NGS

Total RNA was extracted from single expanded blastocysts using the PicoPure® RNA isolation Kit (Thermo Fisher Scientific, MA, USA), according to the manufacturer’s instructions. The quality of total RNA was assessed using High Sensitivity RNA ScreenTape (Agilent Technologies, Santa Clara, CA, USA). NGS libraries were prepared using the Trio RNA-Seq Library Preparation Kit (Tecan Group Ltd., Switzerland), according to the manufacturer’s instructions. ICSI-derived (*n* = 5) and mICSI-derived (*n* = 5) blastocyst RNA samples were used to generate RNAseq libraries. NGS libraries were sequenced by the Illumina NovaSeq S1 platform using 100 bp paired-end sequencing chemistry, conducted by the Australian Genome Research Facility (VIC, Australia).

### RNAseq data analysis and DEG identification

RNAseq raw fastq files were quality checked by '*fastqc*' (version 0.11.9) [78], and trimming of adapters and quality filtering were then performed by '*fastp*' (version 0.20.1) [79] with options: *–detect_adapter_for_pe*, *-q 20*, *–length_required 30*. Trimmed and quality filtered paired-end reads were aligned to the *Sus scrofa* genome assembly Ensembl release 98 [80] by using '*STAR*' (version 2.7) [81] with default parameters. Gene expression was quantified by counting the number of reads aligned to each Ensembl gene model using '*featureCounts*' (version 1.5.2) [82], and output results were assessed for mapping quality by MultiQC version 1.9 [83]. Summary statistics for the RNAseq data are shown in Additional file 2: Table S7.

DEGs between ICSI- and mICSI-derived blastocysts were identified using the *limma-voom* method (version 3.46.0) [84, 85]. The Trimmed Mean of M-values (TMM) normalisation method from *edgeR* (version 3.32.0) was applied to normalise read counts according to library size differences between samples [86]. PCA was performed to visualise the summary of gene expression for all libraries and identify whether the RNAseq library preparation date contributed to variation in gene expression patterns. Therefore, this was included in the linear mixed model as a covariate and batch effect was successfully corrected in the expression data. Genes with low expression were filtered out prior to DEG analysis, keeping genes with at least 1 count per million (CPM) reads in the three samples. Genes are considered differentially expressed if their FDR (false discovery rate) adjusted *p*-value is < 0.05 (Table 2). DEGs were visualised by volcano plot and heatmap using R packages *ggplot2* [77] and *pheatmap* [87]. Data were entered into Ingenuity pathway analysis (IPA) [88] to determine if any signalling pathway was significantly affected by the DEGs [53]. MicroRNA *ssc-mir-10390* [89, 90] target sites in the *Sus scrofa* genome were predicted by *miRanda* [91] with the options: *-sc* 160 and *-strict*.

### Statistical analysis, functional annotation and graphical visualisation

Comparative statistical analyses for WGBS data were conducted by R package *methylKit* [35] using utilities: '*getCorrelation*', '*clusterSamples*', ' *PCASamples*'. Pearson's correlation was conducted using RStudio. DMRs located in gene promoters and intragenic regions were identified based on the Ensembl version 11.1 database [92]. We defined promoters as regions 2,500 bp upstream and 500 bp downstream of the transcription start site. DMR annotations were obtained by using R package *GenomicRanges* [93]. Gene ontology (GO) enrichment analyses for DEGs and DMRs were performed using the PANTHER classification system [94] and Ensembl gene IDs corresponding to DEG and DMR annotation. DEG and DMR data were integrated and filtered by R package *dplyr* [95] by DEG data value with absolute log2FC > 1 and raw *p*-value < 0.05. Results were visualised by bar plot, histogram, box plot, dot plot and line plot using *ggplot2* [77].

## Supplementary Information

**Additional file 1: Figure S1.** Schematic representation of the production of autologous mICSI-derived blastocysts. **Figure S2.** WGBS data of *Sus scrofa* oocytes (Oc) and ICSI- (IB) and mICSI-derived blastocysts (MB) analysed by the 100-CpG window method and visualized by SeqMonk. **Figure S3.** Comparative analysis of WGBS data sets from *Sus scrofa* oocytes (Oc), ICSI- (IB) and mICSI-derived blastocysts (MB). **Figure S4.** DNA methylation status in each *Sus scrofa* chromosome. **Figure S5**. Longitudinal comparison of DMRs to capture differences in the DNA methylation reprogramming process as a result of mtDNA supplementation. **Figure S6.** Methylation status of imprinted genes (A) *KCNQ1*, (B) *GNAS* and (C) *MEST* in *Sus scrofa* oocytes (Oc) and blastocysts (IB and MB). **Figure S7.** PCA of *Sus scrofa* ICSI- (red) and mICSI- (green) derived blastocyst RNAseq data. **Figure S8.** Volcano plots displaying differential gene expression between *Sus scrofa* ICSI- and mICSI-derived blastocysts. **Figure S9.** Expression of (A) 52 DEGs between ICSI- and mICSI-derived blastocysts (Table 2); (B) genes catalysing cytosine methylation and demethylation; and (C) genes involved in embryonic genome activation presented by heatmap. **Figure S10**. Expression of genes of interest in *Sus scrofa* ICSI- and mICSI-derived blastocysts presented by box plots.

**Additional file 2: Table S1**. Summary statistics from *Sus scrofa* oocyte and blastocyst WGBS data analyses. **Table S2**. A list of DMRs between ICSI- and mICSI-derived blastocysts commonly identified by three DMR callers. **Table S3**. GO enrichment analysis for genomic features related to DMRs in the biological process category, analysed by PANTHER (http://www.pantherdb.org/). **Table S4**. GO enrichment analysis for genomic features related to DMRs in the molecular function category, analysed by PANTHER (http://www.pantherdb.org/). **Table S5**. GO enrichment analysis for genomic features related to unique DMRs in the Oocyte vs ICSI-derived blastocyst comparison. **Table S6**. GO enrichment analysis for genomic features related to DMRs unique in the Oocyte vs mICSI-derived blastocyst comparison. **Table S7**. Summary statistics of RNAseq data for ICSI- and mICSI-derived blastocysts. **Table S8**. Prediction of microRNA *ssc-miR-10390* target genes in the *Sus scrofa* genome determined by *miRanda*. **Table S9**. Mitochondrial gene expression in ICSI- and mICSI-derived blastocysts. **Table S10**. DMRs associated with potential DEGs. **Table S11**. Blastocyst development rates following ICSI and mICSI.

## Data Availability

The datasets supporting the conclusions of this article are available in the NCBI Sequence Read Archive (https://www.ncbi.nlm.nih.gov/sra) under the two BioProject IDs PRJNA752230 and PRJNA777282.

## Abbreviations

5hmC: 5-Hydroxymethylcytosine
5hmU: 5-Hydroxymethyluracil
APOBEC1: Apolipoprotein B mRNA editing enzyme catalytic subunit 1
CGI: CpG island
DEG: Differentially expressed gene
COC: Cumulus–oocyte complex
CPM: Count per million
DMR: Differentially methylated region
DNMT: DNA methyl transferase
EGA: Embryonic genome activation
FOXA1: Forkhead box A1
FDR: False discovery rate
GNAS: Guanine nucleotide binding protein alpha stimulating activity polypeptide
GO: Gene ontology
HBEGF: Heparin-binding EGF-like growth factor
ICR: Imprint control region
ICSI: Intracytoplasmic sperm injection
IGF2R: Insulin-like growth factor 2 receptor
KCNQ1: Potassium voltage-gated channel subfamily Q member 1
log2FC: Log2-fold change
logCPM: Log2-counts per million
lncRNA: Long non-coding RNA
MEST: Mesoderm-specific transcript
mICSI: Mitochondrial supplementation in combination with ICSI
IPA: Ingenuity pathway analysis
MII: Metaphase II
miRNA: MicroRNA
mtDNA: Mitochondrial DNA
PCA: Principal component analysis
NGS: Next generation sequencing
PEG: Paternally expressed gene
POLG: DNA polymerase gamma
RGN: Regucalcin
SFF: Sow follicular fluid
TET: Ten-eleven translocation
TMM: Trimmed mean of M-values
WGBS: Whole genome bisulfite sequencing
