# Supplementary material for: Mitochondrial supplementation of Sus scrofa metaphase II oocytes alters DNA methylation and gene expression profiles of blastocysts
Source: Epigenetics Chromatin. 2022 Apr 15;15:12. doi: 10.1186/s13072-022-00442-x (PMC9013150; doi:10.1186/s13072-022-00442-x)
Supplement: Supplementary file 1 — Additional file 1: Figure S1. Schematic representation of the production of autologous mICSI-derived blastocysts. Figure S2. WGBS data of Sus scrofa oocytes (Oc) and ICSI- (IB) and mICSI-derived blastocysts (MB) analysed by the 100-CpG window method and visualized by SeqMonk. Figure S3. Comparative analysis of WGBS data sets from Sus scrofa oocytes (Oc), ICSI- (IB) and mICSI-derived blastocysts (MB). Figure S4. DNA methylation status in each Sus scrofa chromosome. Figure S5. Longitudinal comparison of DMRs to capture differences in the DNA methylation reprogramming process as a result of mtDNA supplementation. Figure S6. Methylation status of imprinted genes (A) KCNQ1, (B) GNAS and (C) MEST in Sus scrofa oocytes (Oc) and blastocysts (IB and MB). Figure S7. PCA of Sus scrofa ICSI- (red) and mICSI- (green) derived blastocyst RNAseq data. Figure S8. Volcano plots displaying differential gene expression between Sus scrofa ICSI- and mICSI-derived blastocysts. Figure S9. Expression of (A) 52 DEGs between ICSI- and mICSI-derived blastocysts (Table 2); (B) genes catalysing cytosine methylation and demethylation; and (C) genes involved in embryonic genome activation presented by heatmap. Figure S10. Expression of genes of interest in Sus scrofa ICSI- and mICSI-derived blastocysts presented by box plots. [file 13072_2022_442_MOESM1_ESM.pdf]

Title:

Mitochondrial supplementation of *Sus scrofa* metaphase II oocytes alters DNA methylation and gene expression profiles of blastocysts.

Authors:

Takashi Okada[1], Stephen McIlfatrick[1], Nhi Hin[2], Nader Aryamanesh[2,3], James Breen[2], and Justin St. John[1*]

Additional File 1 Contents:

Supplementary Figures

Figure S1. Schematic representation of the production of autologous mICSI-derived blastocysts.

Figure S2. WGBS data of *Sus scrofa* oocytes (Oc) and ICSI- (IB) and mICSI-derived blastocysts (MB) analysed by the 100-CpG window method and visualized by SeqMonk.

Figure S3. Comparative analysis of WGBS data sets from *Sus scrofa* oocytes (Oc), ICSI- (IB) and mICSI-derived blastocysts (MB).

Figure S4. DNA methylation status in each *Sus scrofa* chromosome.

Figure S5. Longitudinal comparison of DMRs to capture differences in the DNA methylation reprogramming process as a result of mtDNA supplementation.

Figure S6. Methylation status of imprinted genes (**A**) *KCNQ1*, (**B**) *GNAS* and (**C**) *MEST* in *Sus scrofa* oocytes (Oc) and blastocysts (IB and MB).

Figure S7. PCA of *Sus scrofa* ICSI- (red) and mICSI- (green) derived blastocyst RNAseq data.

Figure S8. Volcano plots displaying differential gene expression between *Sus scrofa* ICSI- and mICSI-derived blastocysts.

Figure S9. Expression of (**A**) 52 DEGs between ICSI- and mICSI-derived blastocysts (**Table 2**), (**B**) genes catalysing cytosine methylation and demethylation and (**C**) genes involved in embryonic genome activation presented by heatmap.

Figure S10. Expression of genes of interest in *Sus scrofa* ICSI- and mICSI-derived blastocysts presented by box plots.

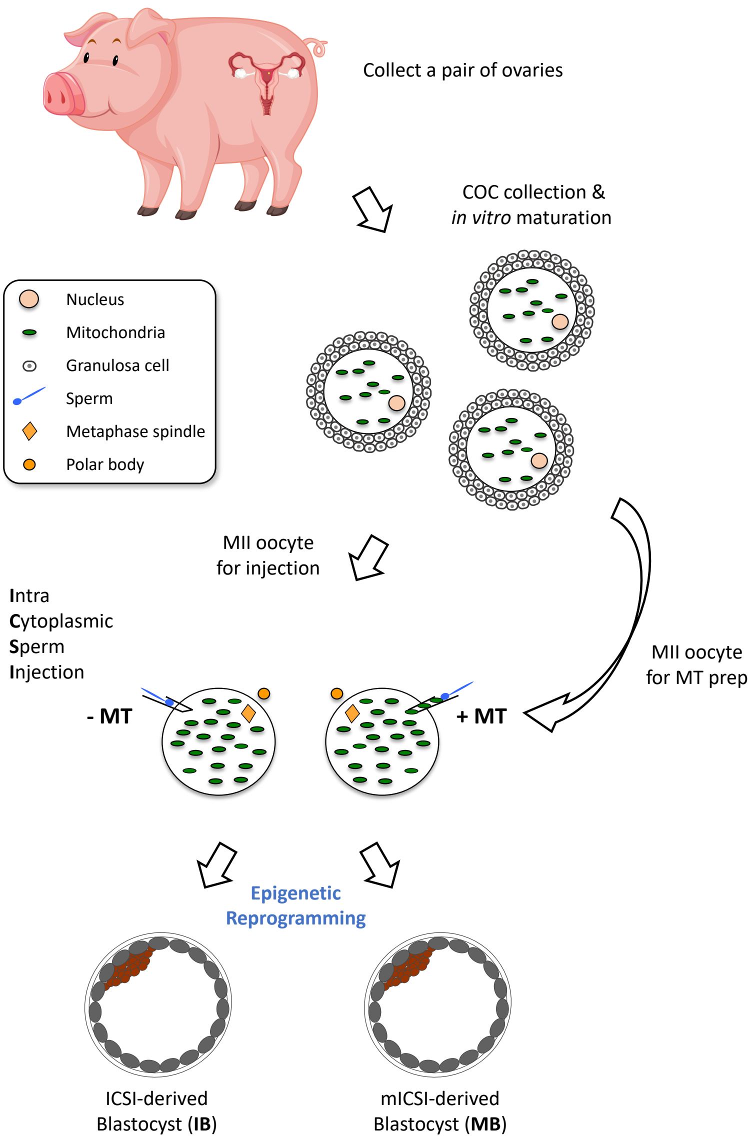

Figure S1. Schematic representation of the production of autologous mICSI-derived blastocysts. Throughout the experiment, each ovary pair collected from an individual pig was treated separately from other ovary pairs. Cumulus-oocyte-complexes (COC) were collected from each ovary pair and cultured *in vitro* to maturation. MII oocytes were prepared from cultured COCs for either i) intracytoplasmic sperm injection (ICSI); or ii) mitochondrial supplementation in combination with ICSI (mICSI). The remaining 'sister' oocytes from each ovary pair were used to prepare the isolate of mitochondria (MT), as indicated by the same colour (green). Consequently, mICSI was performed using mitochondria isolated from sister oocytes to ensure autologous transfer. Blastocysts derived from ICSI or mICSI were collected for DNA and RNA extraction (IB and MB). Images of pig and ovary were designed by brgfx/Freepik.

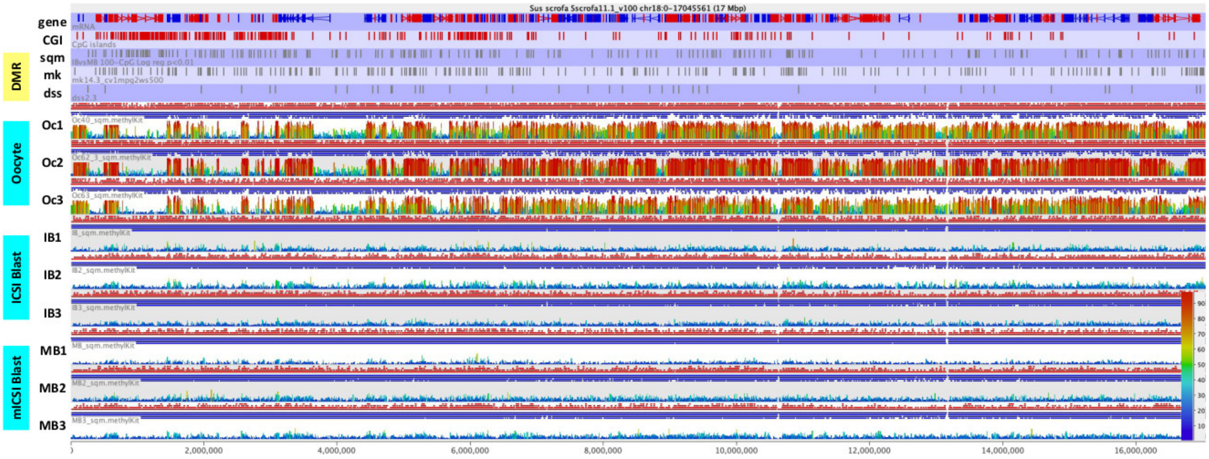

Figure S2. WGBS data of *Sus scrofa* oocytes (Oc) and ICSI- (IB) and mICSI-derived blastocysts (MB) analysed by the 100-CpG window method and visualized by SeqMonk. Levels of CpG methylation were displayed as bar histograms. Scale of levels of methylation are visualized by colour gradient, shown at the bottom right. Red and blue dots above each histogram indicate methylated and unmethylated C counts, respectively. Triplicate BS-seq data for each sample type are shown. Gene and CGI annotated regions are shown at the top. DMRs between ICSI- and mICSI-derived blastocysts from the WGBS data were determined by three DMR callers, SeqMonk (sqm), methylKit (mk) and DSS (dss) and indicated by grey boxes under the genomic features at the top part of the panel.

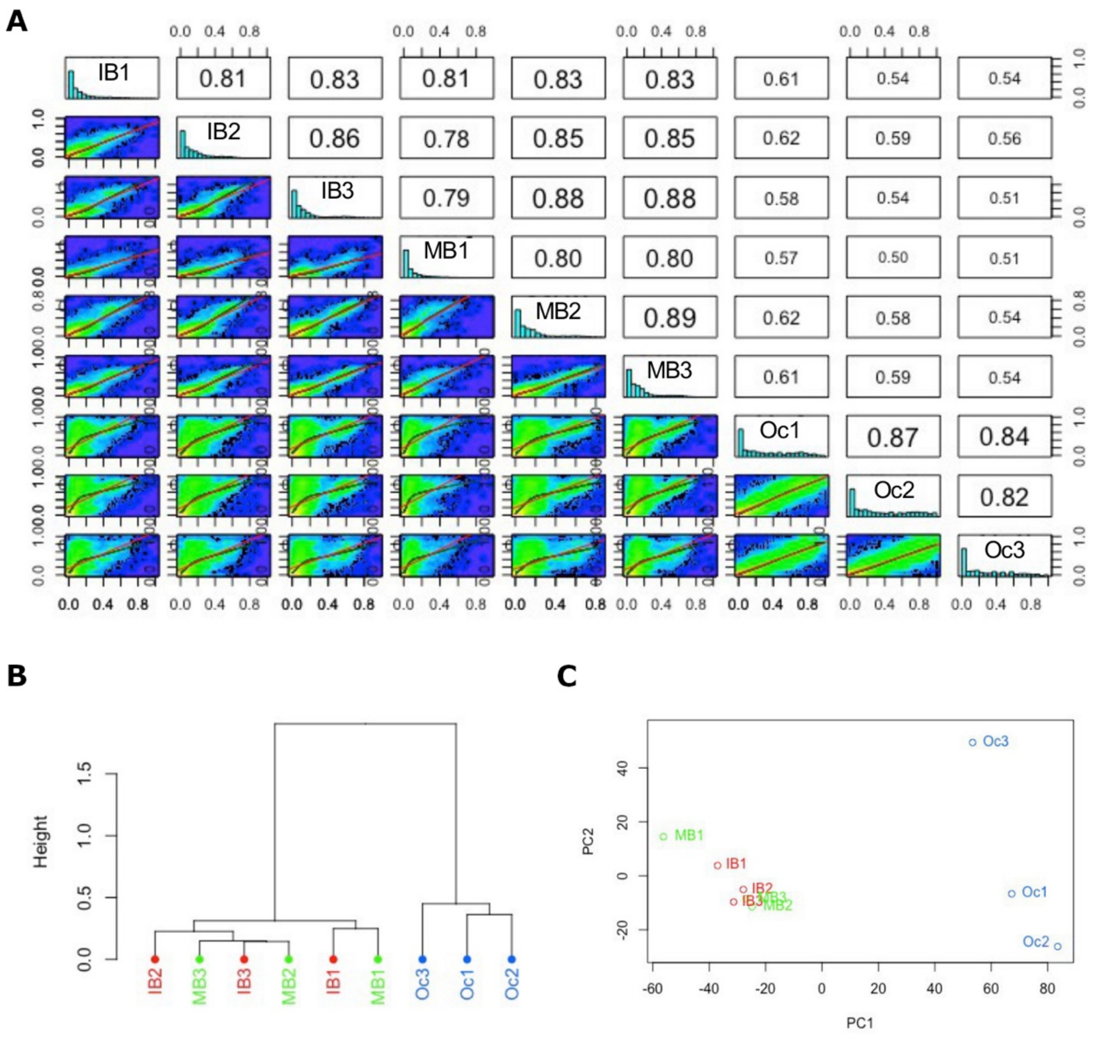

Figure S3. Comparative analysis of WGBS data sets from *Sus scrofa* oocytes (Oc), ICSI- (IB) and mICSI-derived blastocysts (MB). Triplicate BS-seq data were obtained for each sample type. (**A**) Correlation analysis of each WGBS data set. Scatterplots show the relationship between samples containing a fitted line representing linear regression. Pearson's correlation coefficient values for each combination are also presented. (**B**) Hierarchical clustering of BS-seq sample data sets determined by the 'ward' method using correlations as distance measure. (**C**) PCA of BS-seq data sets. All analyses were performed by *MethylKit*.

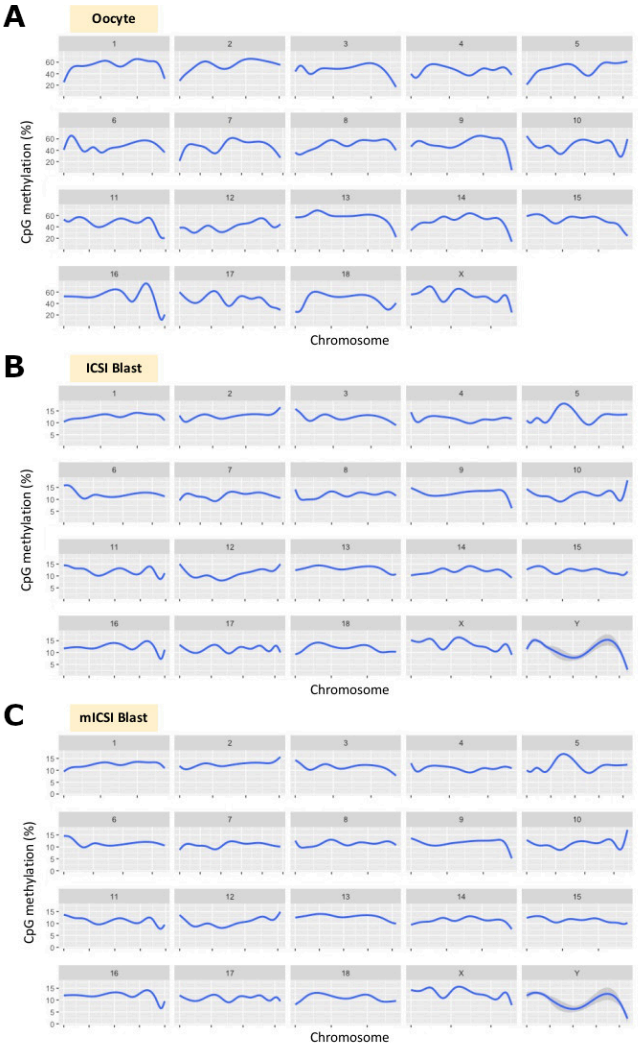

Figure S4. DNA methylation status in each *Sus scrofa* chromosome. (**A**) Oocyte. (**B**) ICSI-derived blastocysts. (**C**) mICSI-derived blastocysts. X-axis represents chromosomal position and y-axis represents levels of CpG methylation (%).

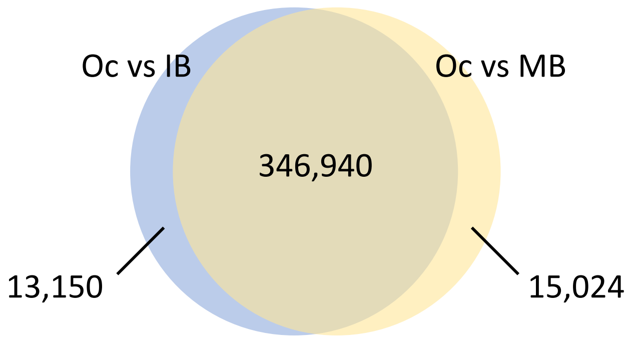

Figure S5. Longitudinal comparison of DMRs to capture differences in the DNA methylation reprogramming process as a result of mtDNA supplementation. Timing of epigenetic reprograming relative to ICSI- and mICSI-derived blastocyst development from oocytes (Oc) is shown in **Fig. S1**. Differences in DNA methylation reprogramming from the oocyte to the blastocyst stage are represented by Venn diagram, showing common and unique DMRs between the two comparisons (Oc vs IB and Oc vs MB). Abbreviations are as shown in Fig. 1.

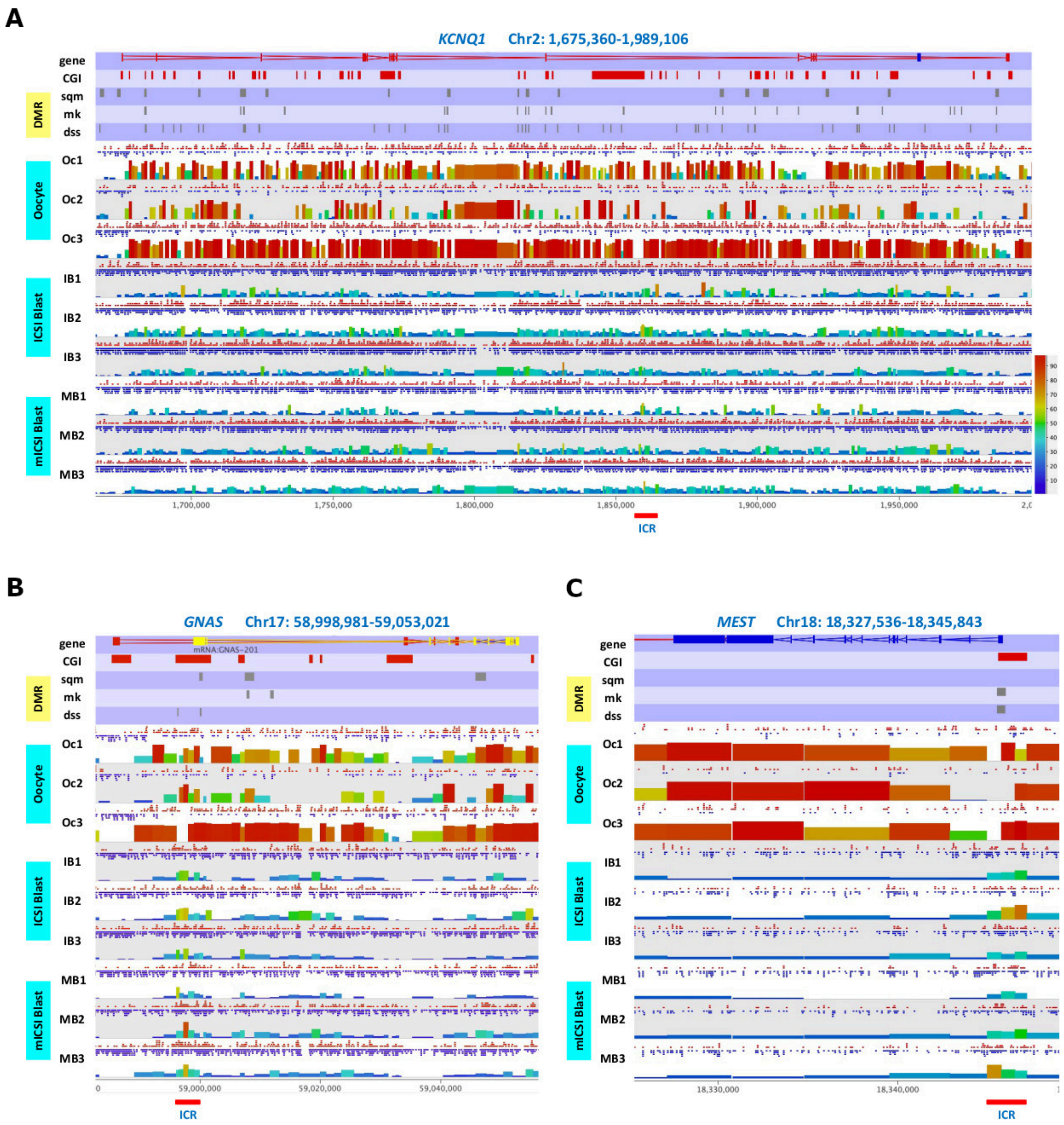

Figure S6. Methylation status of imprinted genes (**A**) *KCNQ1*, (**B**) *GNAS* and (**C**) *MEST* in *Sus scrofa* oocytes (Oc) and blastocysts (IB and MB). Levels of CpG methylation were calculated using the 100-CpG tile method and displayed as a bar histogram in SeqMonk. Red and blue dots above each histogram indicate methylated and unmethylated C counts, respectively. Triplicate BS-seq data for each sample type are shown. Gene and CGI annotated regions are shown at the top. DMR between ICSI- and mICSI-derived blastocysts from WGBS data were determined by three DMR callers, SeqMonk (sqm), methylKit (mk), and DSS (dss), and indicated by grey boxes under the genomic features at the top part of the panel. The genomic region corresponding to the imprint control region (ICR) identified in human and mouse is indicated by the red bar at the bottom of the panel.

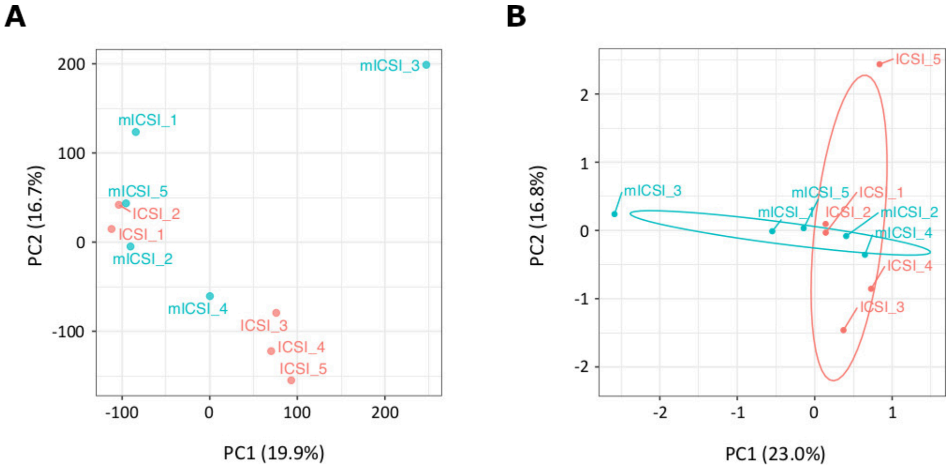

Figure S7. PCA of *Sus scrofa* ICSI- (red) and mICSI- (green) derived blastocyst RNAseq data. (**A**) PCA without adjustment. (**B**) PCA after batch effect correction using library preparation date as a covariate.

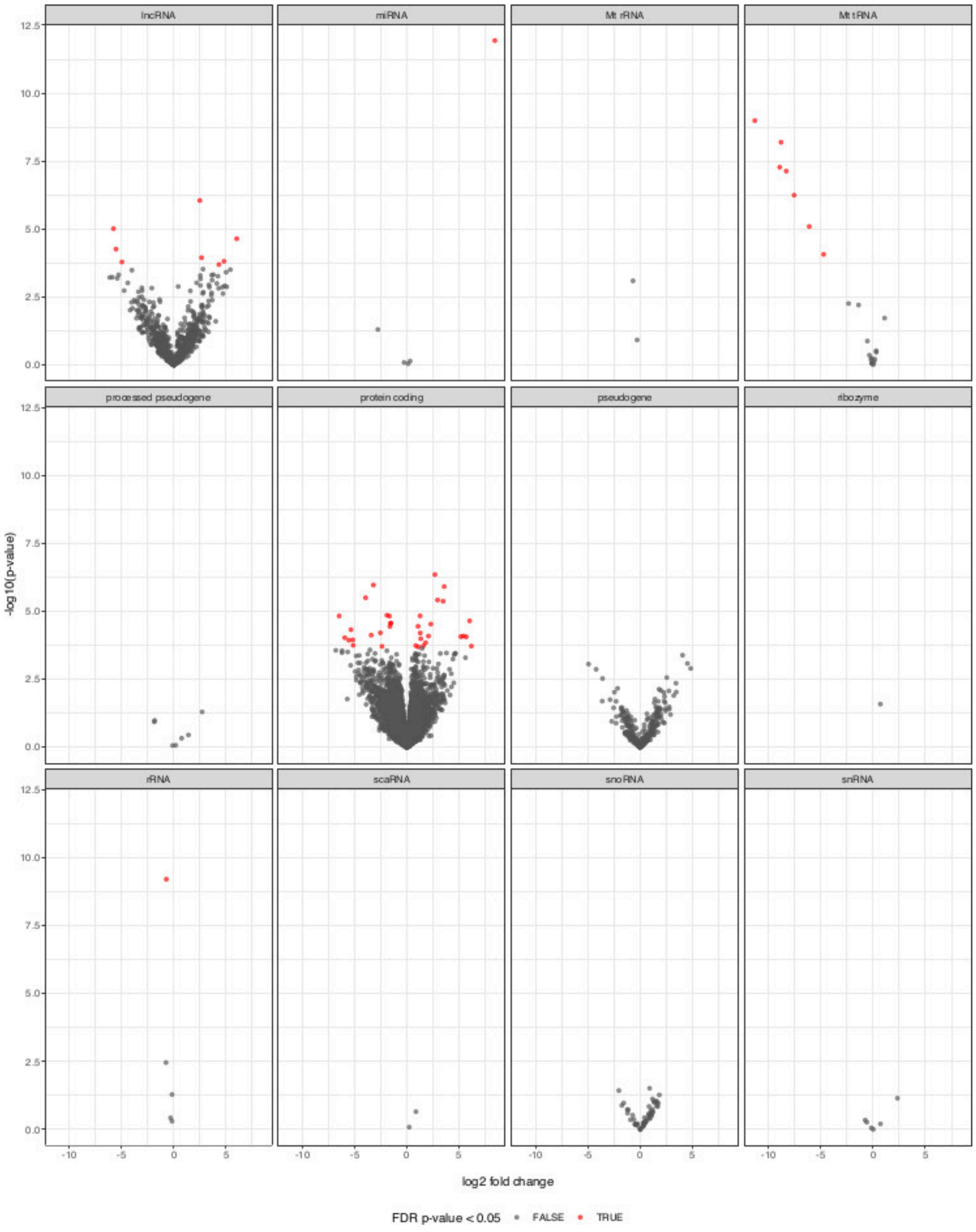

Figure S8. Volcano plots displaying differential gene expression between *Sus scrofa* ICSI- and mICSI-derived blastocysts. The y-axis corresponds to the mean expression value of log10 (*p*-value), and the x-axis displays the log2-fold change value. The red dots represent significant up- or down-regulation in mICSI-derived blastocysts (FDR test *p*-value < 0.05). Positive x-values represent upregulation in mICSI-derived blastocysts against ICSI-derived blastocysts

and negative values represent downregulation. Multiple plots are shown for different types of gene encoding transcripts, including (top row) - lncRNA, miRNA, MT rRNA, MT tRNA, (middle row) - processed pseudogene, protein coding, pseudogene, ribozyme, (bottom row) - rRNA, scaRNA, snoRNA and snRNA.

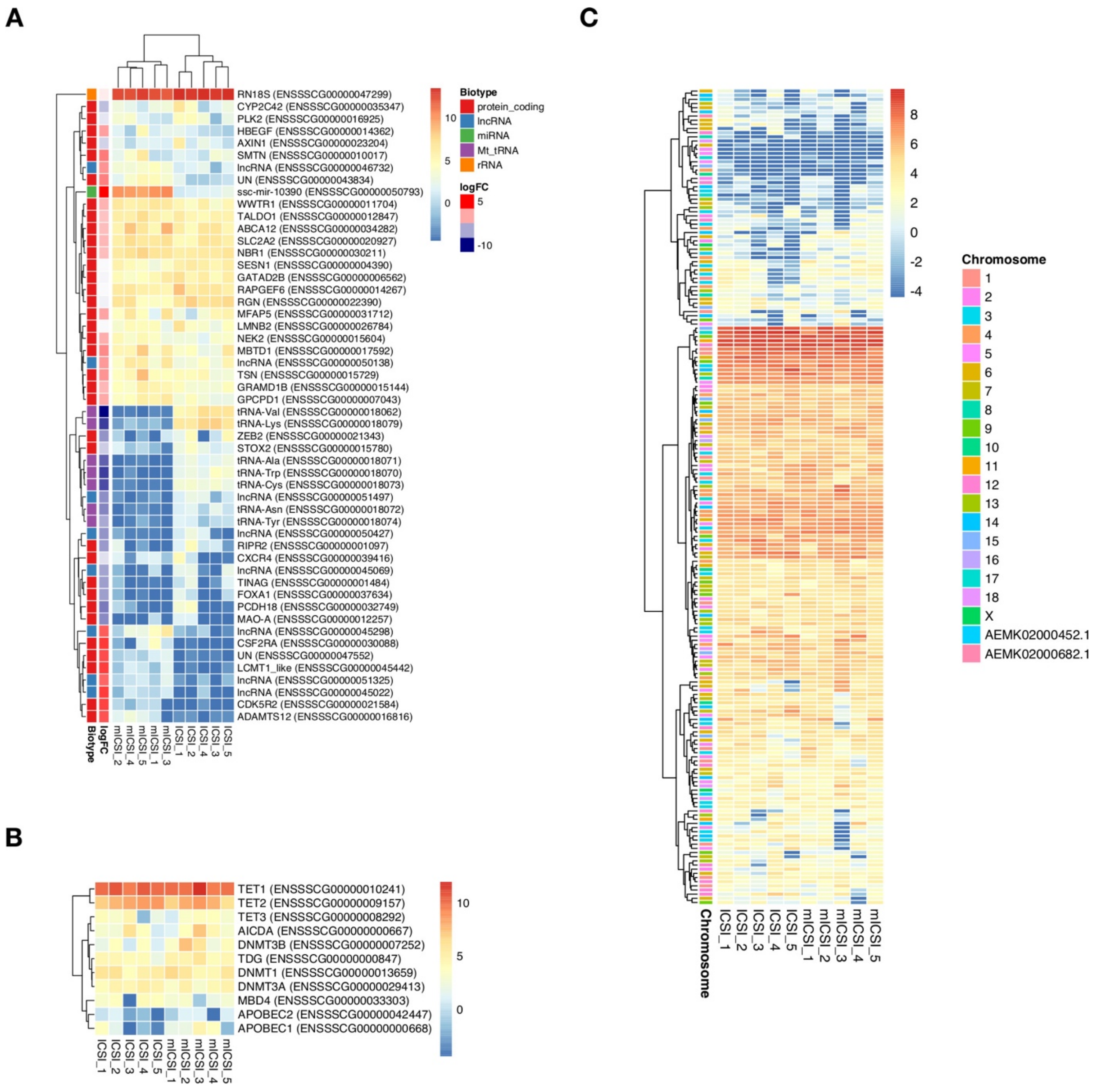

Figure S9. Expression of (**A**) 52 DEGs between ICSI- and mICSI-derived blastocysts (**Table 2**); (**B**) genes catalysing cytosine methylation and demethylation; and (**C**) genes involved in embryonic genome activation presented by heatmap. Rows show individual genes ordered by hierarchical clustering based on gene expression patterns. Each tile in the main matrix represents the levels of expression of a single gene in a single RNAseq data set. Colour of tile indicates levels of expression in TMM-normalized logCPM, and a scale is presented on the right. The biotype of genes, average log2 fold change between ICSI- and mICSI-derived blastocysts in (**A**) and located chromosome in (**C**) are also presented in colour tiles on the left.

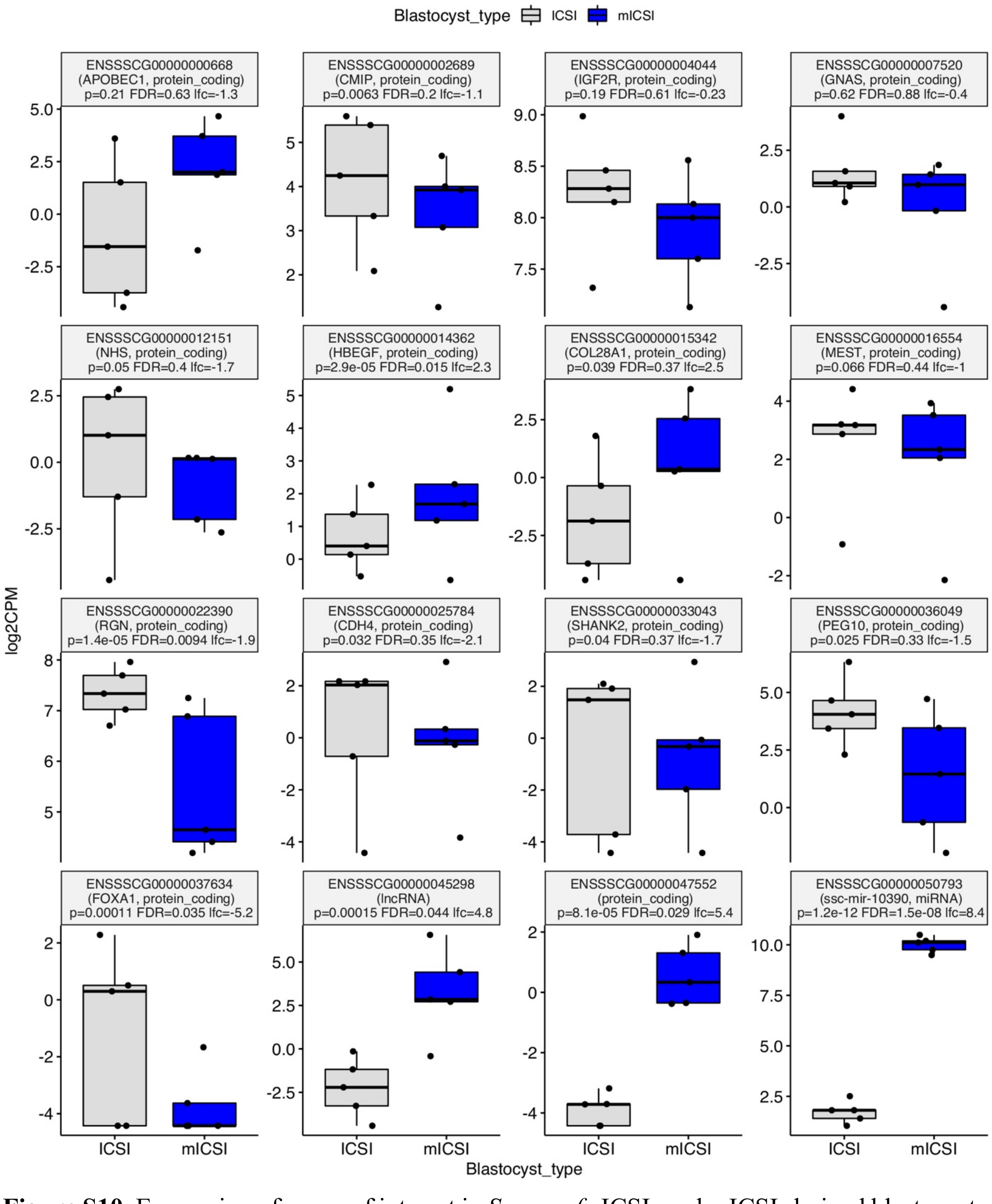

Figure S10. Expression of genes of interest in *Sus scrofa* ICSI- and mICSI-derived blastocysts presented by box plots. X- and Y- axes represent blastocyst type and expression level presented by log2CPM, respectively. Expression values of individual RNAseq sample data are presented by jittered points. Ensembl gene ID, gene symbol, biotype, raw *p*-value (p), false positive discovery rate (FDR) and log2-fold change (lfc) are presented in the title of each graph.
